# *Lantana camara* L. (Verbenaceae) attenuates Ischemia-induced olfactory dysfunction by targeting the α7 nAChR allosteric site and mitigating oxidative-inflammatory cascades in the rat olfactory-memory axis

**DOI:** 10.1016/j.ibneur.2026.06.020

**Published:** 2026-07-01

**Authors:** Symphorien Talom Mabou, Claude Danielle Bilanda, Bernes Rivaldo Tadah Kahou, Ferdinand Tameu Meuladje, Antoine Kavaye Kandeda

**Affiliations:** Department of Animal Biology and Physiology, University of Yaoundé I, P.O. Box 812, Yaoundé, Cameroon

**Keywords:** *Lantana camara*, Ischemia-Reperfusion, Olfactory Dysfunction, α7 nAChR, Kigelinone, Neuroprotection

## Abstract

**Background:**

Olfactory dysfunction is a debilitating yet under-investigated sequela of ischemic stroke, for which no approved pharmacological intervention currently exists. Despite the documented ethnomedicinal use of *Lantana camara* L. in Cameroon for post-stroke impairments, its neuroprotective potential against ischemia-induced olfacto-mnemonic axis damage remains unexplored.

**Purpose:**

To establish a reproducible rat model of post-stroke olfactory dysfunction and evaluate the neuroprotective effects of an aqueous extract of *L. camara* (AELC) against global cerebral ischemia-reperfusion-induced olfacto-cognitive deficits.

**Methods:**

A modified transient global cerebral ischemia-reperfusion model was developed in male Wistar rats via bilateral common and internal carotid artery occlusion. Animals were treated with AELC (140, 280, and 560 mg/kg), minocycline (100 mg/kg), or piracetam (250 mg/kg) as reference compounds. Evaluations encompassed neurological recovery, thermoregulatory profiling, olfacto-cognitive behavioral testing, and biochemical and histological analyses of the olfactory bulb, piriform cortex, prefrontal cortex, and hippocampus. Mechanistic insights were sought through LC-MS phytochemical profiling, *in silico* ADMET analysis, and curvature-based blind molecular docking against the α7 nicotinic acetylcholine receptor (nAChR; PDB: 7KOX).

**Results:**

Ischemia-reperfusion induced severe thermoregulatory failure, locomotor deficits, and significant olfactory dysfunction (Hedges' g > 2.00), correlating with acetylcholine depletion, oxidative stress (elevated MDA and nitrites; depleted GSH), and neuroinflammation (elevated IL-1β and TNF-α). Histological examination confirmed significant neuronal pyknosis, ghost cell formation, and architectural disorganization across the olfacto-mnemonic axis. AELC at 280 and 560 mg/kg effectively reversed these deficits, restoring cholinergic tone (p < 0.001) and preserving neuronal microarchitecture comparably to reference compounds. LC-MS and *in silico* analyses identified Salvigenin and Kigelinone as promising bioactive leads with favorable drug-likeness and high predicted membrane permeance. Kigelinone exhibited predicted binding interactions at a site topographically consistent with known allosteric modulators of α7 nAChR, forming a hydrogen bond with Ala262 of the M2 transmembrane helix, a mechanistic hypothesis warranting functional validation.

**Conclusion:**

These findings establish a reliable rat model of post-stroke olfactory dysfunction and demonstrate that *L. camara* confers significant neuroprotection by modulating the cholinergic-antioxidant-neuroinflammatory axis, validating its ethnomedicinal use and offering a promising natural scaffold for the development of targeted therapeutics against ischemic brain injury.

## 1 Introduction

Stroke is a leading cause of mortality and neurological disabilities globally, with over 100 million victims. It can be hemorrhagic or ischemic (Kamgang et al., 2021). The prevalence of stroke is disproportionately rising in developing countries, driven by suboptimal socioeconomic conditions and limited access to specialized healthcare. Olfaction is the sensory modality responsible for odor detection, identification, discrimination, and recognition, and is indispensable for environmental navigation and adaptation (Silkis, 2023). However, this sense is particularly susceptible to disruption following cerebral ischemia. Post-stroke olfactory dysfunction is common, with reported prevalence rates ranging from 20% to over 70% depending on the population and assessment method employed (Bailliard et al., 2025, Chamberlin et al., 2025, Wehling et al., 2015). Beyond a simple sensory deficit, this impairment significantly affects nutrition, emotional wellbeing, cognitive performance, and overall quality of life in stroke survivors (Bailliard et al., 2025, Chamberlin et al., 2025, Wehling et al., 2015). Olfactory structures such as the olfactory bulbs and piriform cortex are reported to be vulnerable to ischemic injury due to their elevated metabolic demands and abundant vascularization. Consequently, reduced cerebral blood flow compromises these regions, impairing neuronal integrity and disrupting odor processing and recognition (Jiménez et al., 2024). Furthermore, olfaction is uniquely connected to memory, as the olfactory system projects directly to the prefrontal cortex and hippocampus, structures critically involved in memory formation, emotional processing, and decision-making (Døving, 2019). This provides an explanation why smells can trigger powerful memories and emotions (Silkis, 2023). Ischemic lesions to these structures compromise an individual's ability to recall odor-associated memories or utilize olfactory information for adaptive behavior (Daniels and Vermetten, 2016). Beyond direct ischemic insult, secondary pathological processes including acetylcholine depletion, neuroinflammation, and oxidative stress compromise olfactory neurons and exacerbate olfactory dysfunctions(Kondo et al., 2020; Kuriakose and Xiao, 2020).

Several animal models are employed in investigations concerning stroke among which transient occlusion of cerebral arteries is widely used (Li and Zhang, 2021). This procedure effectively mimics the pathological changes observed during ischemic attacks. Cerebral reperfusion injury occurs when blood flow is restored to the brain after a period of ischemia (Kuriakose and Xiao, 2020). Despite its critical role in preventing tissue necrosis, reperfusion can paradoxically exacerbate tissue damage by triggering oxidative stress and inflammation (Khoshnazar et al., 2020). These processes are reported to release free radicals, pro-inflammatory markers, cell death, and long-term neurological sequelae (Li and Zhang, 2021). Emerging neuroanatomical literature suggests that the common and internal carotid arteries supply the olfactory bulbs, piriform cortices, and various cortical and subcortical structures crucial for olfacto-memory (Tahir et al., 2019, Vrselja et al., 2014). Reduced perfusion of these structures is therefore hypothesized to contribute to post-stroke olfactory deficits. While this neuroanatomical link is proposed, practical investigations confirming the hypothesized reduction in anterior cerebral artery blood flow and its subsequent impact on olfactory function in rats are rare. In 1984, Nakashima *et al*. reported a resistance of the olfactory bulb to cerebral ischemia after one-hour unilateral occlusion of the carotid artery in gerbils (Nakashima et al., 1984). That study, however, failed to account for the compensatory role of collateral basilar circulation and the intrinsic regenerative capacity of olfactory sensory neurons within the olfactory bulb. Hwang *et al.* reported a neuronal loss in the glomerular layer of the olfactory bulb after transient ischemia in gerbils (Hwang et al., 2004). However, this research induced transient global cerebral ischemia through a 5-minute bilateral occlusion of the common carotid artery, did not behaviorally characterize this condition, and did not investigate pathological changes in other critical parts of the olfacto-mnemonic system.

Despite the clinical availability of thrombolytic agents such as intravenous tissue plasminogen activator and surgical interventions like thrombectomy, their efficacy remains severely constrained by narrow therapeutic windows and a critical shortage of specialized stroke units in developing nations (Kamgang et al., 2021). Furthermore, the management of stroke-related neurological sequelae persists as a formidable challenge in low- and middle-income regions, necessitating the investigation of accessible, biologically compatible therapeutic alternatives. *Lantana camara* Linn. (Verbenaceae), a hardy, perennial aromatic shrub native to the American tropics and now naturalized across global subtropical regions, represents a promising candidate for such interventions (Chitapalli and Kemisetti, 2024). Botanically, *L. camara* is characterized by quadrangular, often prickly stems, ovate serrated leaves with a scabrous texture, and distinctive umbellate corymbs that undergo floral color transitions to facilitate pollinator signaling (Kato-Noguchi and Kato, 2025). While the species is historically recognized for its traditional use against various neurological pathologies (Dervash et al., 2025), recent ethnobotanical surveys conducted in southern Cameroon documented the frequent use of aqueous leaf extracts by traditional healers to manage post-stroke impairments and neuroinflammation (personal communications, 2023–2024). Similar ethnopharmacological applications have been documented in Ghana, Nigeria, and India, particularly concerning cerebral ischemia and brain injury (Chitapalli and Kemisetti, 2024, Dervash et al., 2025). Pharmacologically, *L. camara* has demonstrated significant *in vitro* antithrombin and thrombolytic potential (Mujbaile et al., 2023), properties directly pertinent to the pathophysiology of ischemic stroke (Matta et al., 2015). Previous studies have further validated its antioxidant, anti-inflammatory, antihypertensive, and neuroprotective properties (Amoah et al., 2023, Ghosh et al., 2010, Kandeda et al., 2022, Naz and Bano, 2013). The plant’s rich chemical profile, including neuroprotective flavonoids and triterpenoids, supports its bioactivity against cerebral insults (Ezzat et al., 2020, Kato-Noguchi and Kato, 2025), yet its specific impact on post-stroke olfactory dysfunction remains a critical research gap.

The primary objective of this study was to evaluate the efficacy of *Lantana camara* aqueous leaf extract in mitigating olfactory-cognitive deficits following cerebral insult through a multidisciplinary approach. This was achieved by first establishing and validating a standardized rat model of post-stroke olfactory dysfunction using a 15-minute global cerebral ischemia-reperfusion protocol. Subsequently, the therapeutic potential of the extract was quantified via behavioral assessments, including buried food-seeking, novel odor recognition, and spatial odor memory tests, to evaluate improvements in olfactory perception and cognitive function. The underlying mechanisms were explored through biochemical profiling of the oxidative-inflammatory surge and cholinergic restoration across the olfactory-memory axis, complemented by semi-quantitative histopathological characterization of olfacto-mnemonic axis. Finally, *in silico* computational modeling via drug-likeness prediction, toxicity profiling, and allosteric modulator identification was utilized to identify key bioactive scaffolds responsible for the observed effects.

## 2 Materials and methods

### 2.1 Animals and ethics

The experiment involved male Wistar rats, aged 8 and 10 weeks, with weight ranging between 150 and 180 g. The animals were housed at the University of Yaoundé I's Laboratory of Animal Physiology under controlled conditions. This included a 12-hour light/dark cycle with temperature maintained between 24°C and 26°C. Standard soy-free laboratory chow and tap water were constantly available. All procedures involving the animals adhered to the ethical guidelines of the Institutional Ethics Committee, under the authority of the Cameroon Ministry of Scientific Research and Technological Innovation (Reg. no. FWA-IRD 0001954, 04/09/2006). These guidelines are in accordance with the U.K. Animals (Scientific Procedures) Act, 1986 for the protection of animals used for scientific purposes. The design of the study, including the allocation of animals to groups, dosages used in the experiment, outcome measures, and statistical methods employed, followed the ARRIVE Guidelines 2.0 (link: https://nc3rs.org.uk/our-portfolio/arrive-animal-research-reporting-vivo-experiments).

### 2.2 L. camara identification, preparation of the aqueous extract, and dosage

A specimen *of Lantana camara* collected in Yaoundé, Cameroon. The plant was identified at the National Herbarium of Cameroon under voucher number 30440/HNC/Cam. The scientific name was confirmed using an online resource (https://www.worldfloraonline.org/taxon/wfo−0000223016#local).

The plant aqueous extract was prepared in accordance with the traditional healer's instructions. The harvested leaves were washed, dried in the shade for 30 days, and then ground into a powder using a mechanical blender (model TT-I777). One hundred grams (100 g) of this powder was decocted in 5 liters of distilled water for 20 min, followed by a 24-hour maceration period at room temperature. The resulting mixture was then filtered using Whatman No. 3 filter paper. The obtained filtrate was subsequently dried in a hot air oven at 40 °C. This process yielded a dry mass of approximately 17 g, corresponding to an extraction yield of 17%.

The doses used in this study were calculated based on the ethnomedicinal information provided by a traditional healer. The healer typically prepares a decoction by boiling two handfuls of leaves in one liter of tap water for approximately 20 min. He prescribed two full glasses (approximately 500 ml total) of this decoction, administered once daily to an adult for two weeks. This traditional preparation procedure was mimicked in the laboratory. Five hundred milliliters (500 ml) of the extract were prepared and subsequently dried in a hot air oven at 40 °C, yielding a dry mass of approximately 2.62 g. This quantity was then normalized to an adult male human equivalent dose (HED) by dividing it by 70 kg, which represents the approximate average body mass of an adult male. Thus, the calculated human equivalent dose was 37.31 mg/kg. The animal dose (AD) of the plant extract was calculated using an interspecies dose conversion formula. This formula, adapted from Nair and Jacob (Nair and Jacob, 2016), expresses the Animal Dose (AD; mg/kg) as the product of the Human Equivalent Dose (HED; mg/kg) and a conversion factor of 6.17. Consequently, the AD was determined to be approximately 280 mg/kg. To explore a dose-response relationship, this determined dose was then flanked by two additional doses: a lower dose of approximately 140 mg/kg (half the primary AD) and a higher dose of approximately 560 mg/kg (double the primary AD). These solutions were administered to the rats orally at a volume of 10 ml/kg.

The selected experimental doses (140, 280, and 560 mg/kg) fall well within the safe therapeutic window for this plant, as observed in our previous studies (Kandeda et al., 2022, Talom et al., 2024). While organic solvent fractions (e.g., methanolic extracts) can exhibit toxicity profiles closer to 2000 mg/kg (Dervash et al., 2025), published toxicological evaluations of crude aqueous leaf extracts of *Lantana camara* using OECD guidelines demonstrate no significant physical, hematological, or histopathological toxicity at acute oral limits up to 2000 mg/kg.

### 2.3 Reference drugs and other chemicals

Post-stroke olfactory dysfunction remains an understudied sequela with no approved pharmacological treatment currently available, underscoring the need for reference compounds with established neuroprotective and neurodegenerative profiles in stroke research. Two compounds were used as reference drugs in the current study: minocycline and piracetam. Minocycline (from Sigma-Aldrich, Hamburg, Germany) exerts pleiotropic neuroprotective effects primarily through inhibition of neuroinflammation, microglial activation, and apoptosis. Given that early post-stroke neuroinflammation is a key contributor to olfactory bulb and cortical olfactory pathway injury, minocycline represented a mechanistically relevant reference compound for attenuating inflammation-driven olfactory impairment (Lu et al., 2025, Zhao et al., 2023). Piracetam (from Sigma-Aldrich, Hamburg, Germany) is reported to improve neuronal membrane fluidity, cerebral blood flow, and neurotransmission, with its strongest clinical evidence documented in the subacute and chronic phases. Piracetam demonstrated benefit in aphasia and cognitive sequelae, supporting its utility as a reference for post-stroke sensory and cognitive recovery outcomes (Güngör et al., 2011, Kessler et al., 2000). Ketamine was purchased from Sigma-Aldrich, Hamburg (Germany); trichloroacetic acid and thiobarbituric acid from Rhone-Poulenc laboratories, Lyon (France); and diazepam from La Roche laboratories (Switzerland). Additionally, Ellman reagent, adrenaline, formalin, and other reagents were purchased from Sigma Chemical Co., St. Louis (United States).

### 2.4 Experimental timeline and procedures

**Fig. 1:**
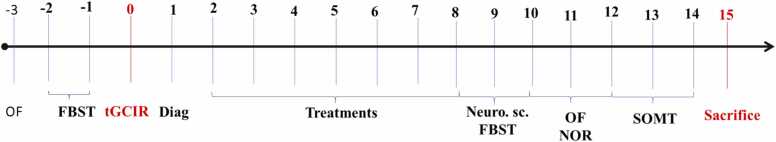
Timeline of experiment FBST, food buried-seeking test, TGCIR, Transient global ischemia and reperfusion; Diag, diagnostic; Neuro. Sc., Neurological score; OF, Open field test; NOR, Novel odor recognition test; SOMT, spatial odor memory test.

To establish a baseline olfactory performance and exclude animals with pre-existing olfactory deficits, a buried food-seeking and the open field tests were administered three days (days −3, −2, and −1) before the experimental induction of transient global cerebral ischemia and reperfusion (IR). On the experimental day (day 0), animals underwent either IR via bilateral common and internal carotid arteries occlusion or sham surgery. On the following day (day 1), a comprehensive neurological assessment, including neurological scoring, buried food seeking, and open field test, was conducted. Animals that didn’t exhibit neurological deficits with olfactory dysfunction were excluded from the study. The remaining animals received daily treatment for seven days (days 2–8). Behavioral assessments (FBST and OF) were repeated on days 8 and 9 to evaluate treatment efficacy. To assess short-term memory impairments, novel odor recognition (NOR) and spatial odor memory tests (SOMT) were performed from days 9–14. Finally, on day 15, animals were euthanized for subsequent biochemical and histological brain analyses (Fig. 1).

### 2.5 Induction of global ischemia and reperfusion

Global cerebral ischemia with Olfactory dysfunction was induced by transient bilateral occlusion of the common and internal carotid arteries. Twelve hours before surgery, the rats were fasted. On the day of the experiment, the rats were anesthetized with ketamine (80 mg/kg, i.p.) and diazepam (5 mg/kg, i.p.). The anesthetized rats were placed on their backs on a surgical board. The surgical area (neck) was thoroughly disinfected with iodine and 75% alcohol. A midline incision was made in the lower neck using a surgical blade and scissors. The submandibular gland was carefully displaced using forceps to expose the sternocleidomastoid and sternohyoid muscles. The common and internal carotid arteries were meticulously separated from surrounding tissues using forceps, taking great care to avoid injuring the vagus nerve. Both arteries were securely ligated with 4–0 silk sutures placed side by side. A successful occlusion was visually confirmed by the cessation of arterial pulsation and blanching of these vessels. These ligatures remained in place for 15 min. Afterward, the surgical incision was sutured closed with silk sutures. The rats were then returned to their cages and monitored until full recovery from anesthesia. Internal carotid artery (ICA) occlusion was specifically targeted to prevent collateral perfusion via the external carotid arteries, thereby ensuring more uniform and extensive neuronal damage across experimental groups.

Body temperature was used as an indirect physiological parameter to indicate successful ischemia induction. Temperature measurements were systematically recorded at several critical intervals:

- **Baseline temperature (T0):** This measurement was obtained immediately before the commencement of the surgical procedure;
- **Intra-procedure temperature (T10):** A subsequent measurement was taken 10 min into the surgical procedure;
- **End-of-procedure temperature (T15):** The final intraoperative temperature was recorded at the conclusion of the surgical procedure;
- **Post-operative temperatures (T30 and T60):** To assess recovery and thermal regulation, additional measurements were taken 15 min (T30) and 30 min (T60) after the completion of the surgical procedure.

### 2.6 Rectal temperature measurement procedure

To ensure a robust and easily comparable assessment of temperature variation during surgical procedures, a cohort of eight (08) sex- and age-matched rats was included. These rats did not receive anesthesia nor undergo any surgical procedure, and thus served as the normothermic control group. Dangarembizi *et al.*’s protocol was used to evaluate rectal temperature (Dangarembizi et al., 2017). This temperature was meticulously measured using a digital thermometer (model iProven DTR−1221A) at various time points throughout a surgical procedure. Initially, for each rat, gentle but firm restraint was achieved by employing a towel wrap, which effectively immobilized the animal while minimizing stress. Vaseline was then applied to the tip of the thermometer probe to ensure smooth and atraumatic insertion into the rectum. The anus was carefully located at the base of the tail. Next, the thermometer probe was then gently inserted into the anus to a depth of approximately 2–3 cm. This depth was chosen to ensure accurate measurement of core body temperature. Once the probe reached the correct depth, it was held steadily in place to allow for thermal equilibration. Finally, after an approximate 10-second stabilization period, the temperature value displayed on the digital thermometer was recorded. Following each measurement, the thermometer probe was immediately and thoroughly disinfected with 70% alcohol to maintain hygiene and prevent cross-contamination.

### 2.7 Selection, grouping, and treatments

**Fig. 2:**
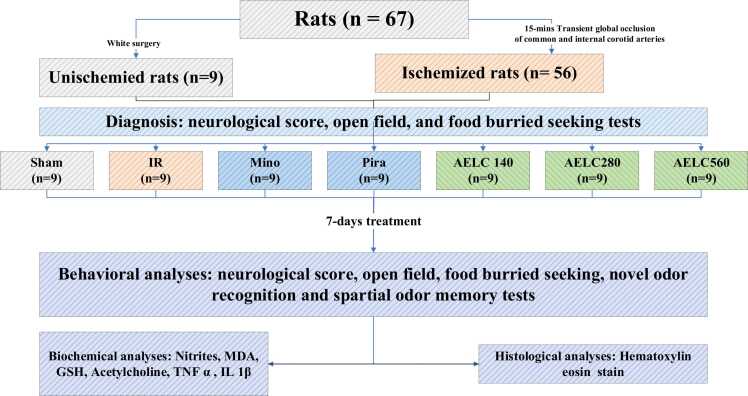
Schematic of the study design including all regimens.Sham, sham-operated group treated with distilled water (10 ml/kg, *p.o*.); IR, Ischemia-reperfusion group treated with distilled water (10 ml/kg, *p.o*.); Mino, minocycline-treated group (100 mg/kg, *p.o*.); Pira, piracetam-treated (250 mg/kg, *p.o*.); AELC (140, 280, 560), aqueous extract of *Lantana camara* treated at 140, 280, 560 mg/kg, *p.o*.).

Animals were initially screened based on intraoperative thermal regulation. Animals exhibiting a significant decrement in core body temperature compared to the average sham control group’s temperature at 10 and 15 min were included for behavioral analysis. Following surgical recovery, all animals, including those from the sham control group, were subjected to a battery of neurological deficit assessments to confirm successful cerebral ischemia induction and associated functional impairments. These tests included the modified Neurological Severity Score (mNSS), the open field test, and buried food-seeking task. Olfactory dysfunction was operationally defined as a latency to locate buried food exceeding the sham group mean by more than one standard deviation. Only animals meeting this criterion, in addition to demonstrating confirmed neurological deficits on the mNSS and open field test, were eligible for randomization into treatment groups (Fig. 2). A total of 56 animals underwent surgery; two animals did not exhibit olfactory dysfunction and were excluded from the treatment arms based on pre-defined exclusion criteria. These treatment regimens included:

• An **IR group** (negative control) was treated with distilled water (10 ml/kg, *p.o*);
• **Two positive control groups** were treated with piracetam (200 mg/kg, *p.o*.) or minocycline (100 mg/kg, *p.o*.);
• **Three test groups** received an aqueous extract of *L. camara* (140, 280, and 560 mg/kg, *p.o*.);
• **The sham-operated group** was added and treated with distilled water (10 ml/kg, *p.o*.).

Treatments were administered once daily for seven days. After these treatments, the rats were subjected again to the same behavioral tests. Novel odor recognition and spatial odor memory tests were also conducted to assess odor memory (Fig. 2).

### 2.8 Behavioral analyses

#### 2.8.1 Neurological deficits score

An 18-point Modified Neurological Severity Score (mNSS) was used to assess neurological deficits in the rats, up to a maximum of 18, indicating more severe neurological impairment. The evaluation is categorized into four main components: Motor Tests (6 points), which assess limb flexion and head orientation when the rat is raised by the tail, as well as gait and stability when placed on the floor; Sensory Tests (2 points), which evaluate visual, tactile, and proprioceptive responses; Beam Balance Tests (6 points), which grade the animal's ability to maintain posture or grasp a beam, ranging from steady balance to immediate falls; and Reflexes and Abnormal Movements (4 points), which test the pinna, corneal, and startle reflexes while also monitoring for seizures or myodystony (Ruan and Yao, 2020).

#### 2.8.2 Open field test

The open-field test was employed to assess the rats' exploratory behavior and general reactivity to a novel environment (Seibenhener and Wooten, 2015). The apparatus used was made up of wood (L: 100 × W: 100 cm × H: 45 cm). The floor was divided into 17 equal squares (10 ×10 cm): 16 squares comprised the interior, and one square represented the center. To reduce neophobic responses, the rats were acclimatized in their home cages for an hour before testing. Each rat was then placed in the center of the open field and allowed to explore freely for 5 min. The following parameters were recorded: distance traveled, average speed, and mobility (ambulatory) time. After each test, the rat was put in the cage, and the apparatus was thoroughly cleaned with 70% ethyl alcohol to remove any residual odors or traces of the previous animal.

#### 2.8.3 Buried food test for olfaction evaluation

Rats were fasted for 12 h before each test. Subsequently, they were individually placed in a new cage with fresh bedding for a 5-minute habituation period. On the following day, a piece of food pellet was buried 3 cm deep and randomly at a corner of the test cage. The animal was then positioned in the opposite corner of the cage and allowed to search for the buried food. The time taken (latency to buried food) for the rat to discover and begin consuming the food was recorded. Rats that could not complete the task in 3 min were awarded a 180 s score. To confirm that the test relied on olfaction rather than vision and locomotion, an additional trial (latency to unburied food) was conducted with the food placed openly on the bedding (Machado et al., 2018).

#### 2.8.4 Novel odor recognition test

The novel odor recognition test is a behavioral assessment used to evaluate an animal's olfactory memory. It operates on the principle that animals tend to investigate unfamiliar scents more than familiar ones (Aqrabawi and Kim, 2018). This test was conducted over three consecutive days, comprising a habituation phase, an encoding phase, and a retrieval phase. During the habituation phase, the animals were placed in an open field and allowed to explore for 5 min. During the encoding phase, the animals were exposed for 10 min to two identical odor sources positioned at opposite ends of the open field. During the retrieval phase, one of the familiar odor sources was replaced with a novel odor, and the animals were allowed to explore the open field for 5 min. The duration of exploration of each odor source was recorded during this phase, and the preference index was subsequently calculated from these exploration duration data. After each trial, the animal was returned to its home cage and the apparatus was thoroughly cleaned with 70% ethanol to eliminate residual odors and olfactory cues from preceding trials.

#### 2.8.5 Spatial odor memory test

The purpose of the spatial odor recognition test was to measure the ability of rats to remember an odor's location within a specific environment (Aqrabawi and Kim, 2018). It was hypothesized that correct memory would lead mice to investigate the odor presented in a novel location within the familiar context. The test consisted of two habituation phases, two encoding phases, and one retrieval phase, each lasting 5 min. During the habituation phases, animals were placed in open field A and, 15 min later, in open field B, to allow familiarization with both environments. Twenty-four hours later, two consecutive encoding phases were performed, one in open field A and one in open field B, with a 15-minute interval between phases. Open field A was configured with two different odor sources positioned at opposite ends of the arena (odor 1 on the left and odor 2 on the right). Open field B was configured with the same odor sources in the reversed positions (odor 2 on the left and odor 1 on the right). During the retrieval phase, two identical odor sources were placed on both sides of the arena, and the exploration duration for each animal was set to 5 min. The total distance travelled, total exploration duration, and discriminative index were subsequently calculated. After each trial, the animal was returned to its home cage and the apparatus was thoroughly cleaned with 70% ethanol to eliminate residual odors and olfactory cues from preceding trials.

### 2.9 Sacrifice, brain dissection, and preparation of homogenates

Rats were sacrificed by cervical decapitation under ether anesthesia. The brains were subsequently extracted, rinsed in 0.9% saline, dried, weighed, and frozen for solidification. Following solidification, the olfactory bulbs, piriform and prefrontal cortices, and hippocampi were dissected from each brain. Each tissue sample was homogenized in 2 ml of Tris buffer (HCl 50 mM; KCl 150 mM; pH 7.4). The homogenate was then transferred to vials and centrifuged at 3000 rpm for 15 min. The resulting supernatants were collected and stored at −20 °C.

### 2.10 Biochemical assays for oxidative and nitrosative stress markers

Levels of oxidative and nitrosative stress markers in the samples were quantified using established spectrophotometric methods. For the determination of malondialdehyde (MDA), a marker of lipid peroxidation, tissue homogenates were deproteinized with 20% (w/v) trichloroacetic acid (TCA) and reacted with 0.67% (w/v) thiobarbituric acid (TBA) at 95°C for 15 min (Wilbur et al., 1949). Following centrifugation at 3000 × g for 10 min, the absorbance of the supernatant was measured at 532 nm, and MDA concentrations (μmol/g) were calculated using the molar extinction coefficient 1.56 × 10^5^ M^−1^ cm^−1^. Nitrite levels (μM), serving as an indicator of nitric oxide production, were assessed utilizing the Griess reaction(Tsikas, 2007). Sample supernatants were mixed with an equal volume of Griess reagent (1% (w/v) sulfanilamide and 0.1% (w/v) N-(1-naphthyl) ethylenediamine dihydrochloride in 5% (v/v) phosphoric acid). The mixture was incubated in the dark at room temperature for 20 min, and the absorbance was recorded at 540 nm. Nitrite concentrations were interpolated from a sodium nitrite standard curve. Finally, reduced glutathione (GSH) content (μmol/g) was determined by measuring the reduction of Ellman’s reagent (Ellman, 1959). TCA-deproteinized sample supernatants were combined with 0.1 M phosphate buffer (pH 8.0) and 5,5′-dithiobis-(2-nitrobenzoic acid) (DTNB). After a 10-minute incubation at room temperature, absorbance was measured at 412 nm, and GSH levels were quantified using a standard calibration curve of pure GSH.

### 2.11 Acetylcholine level assay

In the presence of acetylcholinesterase, acetylcholine is degraded to thiocholine. The reaction of this metabolite with the thiol group of 5,5′-dithiobis−2-nitrobenzoic acid (DTNB) results in the formation of a yellow complex known as thionitrobenzoic acid. The absorbance of the latter at 540 nm was proportional to the concentration of acetylcholine in the medium. The concentration of acetylcholine was determined using the Alm and Augustinson method (Alm, AUGUSTINSSON, 1957). 0.8 ml of homogenate was placed in dry vials, then added 1.4 ml of distilled water, 0.2 ml of physostigmine (1.5 mM), and 0.8 ml of TCA (1.84 M). After centrifuging the mixture, 1 ml of the supernatant was removed and introduced into new dry vials containing 1 ml of basic hydroxylamine. This new mixture was incubated for 15 min at 25°C before 0.5 ml of HCl (4 M) and 0.5 ml of FeCl3 (0.37 M) were added. Finally, the absorbance was read at 540 nm against a blank using a spectrophotometer, and the acetylcholine content was expressed in µmol/g wet tissue weight according to the equation:

### 2.12 IL-1β and TNF-α levels assay

Quantitative assessment of these cytokines was performed using commercially available ELIZA kits. Its principle is based on sandwiching the antigen (the protein of interest, such as IL-1β, TNF-α) between two specific antibodies. A primary antibody (capture antibody) is initially attached to the bottom of the wells. After antigen binding and washing, a secondary antibody (detection antibody) is added to the reaction medium. This secondary antibody is conjugated with biotin, which enables revelation by streptavidin-conjugated peroxidase. The assay procedure begins by attaching specific primary antibodies 10 µg for each protein to the bottom of dry vials via adsorption, followed by a 24-hour incubation at 4°C. Each vial is then washed five times with wash buffer. Afterward, 50 µl of specific assay diluent solutions (RD1–21 for IL-1β and RD1–42 for TNF-α) are added to each tube. Next, a specified volume of Tris-buffer (HCl 50 mM; KCl 150 mM; pH 7.4) is added to the blank/zero standard vials, and a given volume of homogenate is added to the test tubes. This volume was 50 μl for IL1-β and TNF-α. All tubes were incubated at 37°C for 2 h and subsequently washed five times with wash buffer. Then, biotin-conjugated detection antibody specific for each protein is added (100 μl for IL1-β and TNF-α). The vials are incubated a second time at 37°C for 2 h before another five washes with wash buffer. Subsequently, 100 μl of peroxidase-coupled streptavidin conjugate is added to each tube. Tubes were then incubated for 30 min at room temperature, protected from light. The enzymatic reaction is stopped by adding 100 μl of stop solution (H_2_SO4, 0.5 M). Finally, the absorbance was read at 450 nm against the blank using a spectrophotometer, and cytokine concentrations are expressed in ρg/ml.

### 2.13 Histological preparation and microarchitectural assessment

The study employed a standardized histological protocol adapted from Woods *et al.* (Woods et al., 2019) to analyze the microarchitecture of the olfactory bulb (OB), anterior and posterior portions of the piriform cortex (PC), prefrontal cortex (PFC), CA1, CA3, and dentate gyrus of the hippocampus in the brain of rats. The findings derived from this sample size should be interpreted as preliminary and descriptive in nature, intended to support mechanistic plausibility rather than to establish quantitative efficacy. Following sacrifice, brains (n = 4 per group) were carefully extracted and post-fixed in 4% paraformaldehyde for 24 h at 4°C. Tissue blocks were subsequently dehydrated through a graded ascending ethanol series (70%, 80%, 90%, 95%, and 100%), cleared in xylene (two changes, 30 min each), and embedded in paraffin. Coronal 5 µm) were obtained using an Auxilab/Nahita ZFP010 Basic Rotary Microtome, mounted on poly-L-lysine-coated glass slides, and dried overnight at 37°C. Sections were collected at standardized stereotaxic coordinates targeting seven brain subregions in accordance with the Paxinos and Watson Rat Brain Atlas (7th edition, 2014). Mounted sections were deparaffinized in xylene (two changes, 5 min each), rehydrated through a descending ethanol series (100%, 95%, 90%, 80%, and 70%), rinsed in distilled water before staining. Sections were stained with hematoxylin and eosin (H&E) following standard protocols. Briefly, slides were immersed in Mayer's hematoxylin for 5 min, differentiated in 1% acid alcohol, blued under running tap water for 5 min, counterstained with eosin Y (1%) for 2 min, dehydrated through an ascending ethanol series, cleared in xylene, and cover slipped with DPX mounting medium. All stained sections were examined at 250 × magnification using a Scientico STM−50 optical microscope equipped with a digital camera for image capture. Image analysis was subsequently performed using ImageJ software version 1.54i (Schneider et al., 2012).

Histopathological evaluation was conducted independently by two trained raters who were blinded to treatment group allocation throughout the scoring procedure. Four microarchitectural criteria were assessed in each brain subregion using a pre-defined semi-quantitative scoring system. Neuronal density was graded as: 0, normal; 1, mild reduction (< 25%); 2, moderate reduction (25–50%); 3, severe reduction (> 50%). Ghost cell and eosinophilic neuron formation were graded as: 0, none; 1, rare (< 5 per high-power field [HPF]); 2, occasional (5–15/HPF); 3, frequent (> 15/HPF). Neuropil vacuolation (spongiosis) and tissue integrity were each assessed on a 0–3 ordinal scale, where 0 denoted normal appearance, 1 minor disruption, 2 clear disruption, and 3 severe disorganizations. All criteria were scored independently by both raters on the same slide set. Interrater reliability was quantified using Cohen's weighted kappa (κ) for each criterion–subregion combination, computed using R package irr v.0.84. Agreement levels were interpreted according to the benchmarks proposed by Landis and Koch (1977): κ < 0.20, slight; 0.21–0.40, fair; 0.41–0.60, moderate; 0.61–0.80, substantial; and 0.81–1.00, almost perfect agreement. In cases of scoring discrepancy between the two raters, consensus was reached through structured discussion, and the agreed score was adopted for all subsequent analyses.

### 2.14 LC-MS analysis of the aqueous extract of *L. camara*

The LC-MS analysis was executed using an Agilent 1290 Infinity II UHPLC system coupled to a Thermo Orbitrap Exploris 120 mass spectrometer. The separation was achieved on a Waters Acquity UPLC BEH C18 column (100 × 2.1 mm, 1.7 μm), maintained at 40 °C, and samples were contained in certified LC-MS glass vials with PTFE/silicone septa. LC-MS grade water (mobile phase A) and acetonitrile (mobile phase B), both containing 0.1% (v/v) formic acid, were used as the mobile phase. The extract was eluted using a gradient program that began at 5% B and ramped to 95% B over 10 min, followed by a re-equilibration step, all at a flow rate of 0.4 ml/min, for a total run time of 15 min. Ionization was performed using an electrospray ionization source in both negative and positive ion modes, with the capillary voltage set to 2.5 kV; the sheath and auxiliary gas flows were set to 40 and 10 arbitrary units, respectively, with a heater temperature of 300 °C and a capillary temperature of 320 °C. Data were acquired in full scan mode across a mass range of *m/z* 50–1200 using a data-dependent acquisition strategy. Post-acquisition, data were processed using XCMS Online, and peak identification was performed by comparing the detected peaks with data reported in the literature available in the SciFinder database for *Lantana camara.* The analytical run included quality control procedures, in which solvent blanks and system suitability standards were injected before and after the batch, and pooled quality control samples were run every 5 injections to monitor system stability. The acceptance criteria for the analysis were set to a mass accuracy of less than 5 ppm error and a retention time tolerance of ±0.1 min. Semi-quantitative analysis of the LC-MS chromatogram was performed by measuring peak areas using ImageJ software version 1.54i. The relative abundance of each identified compound was expressed as a percentage of the total peak area, calculated as the ratio of individual peak area to the sum of all detected peak areas.

### 2.15 In silico studies

#### 2.15.1 Assessment of physicochemical properties, drug-likeness, and organ toxicity for identified natural compounds

The chemical structures for the identified compounds found in the AELC were retrieved from the PubChem database. To ensure compatibility with computational tools, each structure was converted into its Canonical SMILES string. All molecules were processed in their neutral form at physiological pH to avoid ionization biases in lipophilicity calculations. Physicochemical parameters were computed using the SwissADME web server (Swiss Institute of Bioinformatics). The following descriptors were extracted to assess Lipinski’s Rule of Five (Ro5):

• ***Molecular Weight (MW):*** Calculated as the sum of atomic masses (g/mol).
• ***Lipophilicity (LogP):*** Determined using the MLogP (Moriguchi) topological method to assess the octanol-water partition coefficient.
• ***H-Bond Donors (HBD):*** Defined as the count of hydrogen atoms bonded to nitrogen (N) or oxygen (O).
• ***H-Bond Acceptors (HBA):*** Defined as the count of N or O atoms with available lone pairs.

The Lipinski violation count was recorded based on the criteria: MW ≤ 500, MLogP ≤ 4.15, HBD ≤ 5, and HBA ≤ 10. Permeance was qualitatively estimated based on the BOILED-Egg model (Brain Or IntestinaL EstimateD permeation) and GI absorption scores provided by the platform.

Toxicological endpoints were predicted using the ProTox−3.0 platform, which utilizes machine learning models trained on chemical similarities, pharmacophores, and fragment propensities. Neurotoxicity and hepatotoxicity endpoints were assessed using the platform's multi-class classification models. Predictions were categorized as "Active" or "Inactive" based on the presence of specific toxicophores and structural motifs linked to clinical adverse outcomes. The confidence scores provided by the server cross-referenced with the literature to ensure the reliability of the predictions for each compound's specific chemical class.

#### 2.15.2 Molecular docking protocol

• **Protein and ligands preparation**.

The structural coordinates of the human alpha7 nAChR (PDB ID: 7KOX) were retrieved from the RCSB Protein Data Bank. Protein preparation was conducted using the PlayMolecule platform (ProteinPrepare module). This included the assignment of proper protonation states at physiological pH (7.4), the addition of missing hydrogen atoms, and the optimization of the hydrogen-bond network. Ligand structures for Salviginin (CID: 161271), Kigelinone (CID: 442752), and the reference modulator PNU−120596 (CID: 311434) were similarly processed through PlayMolecule to ensure optimized 3D geometries and appropriate ionization states.

• **Cavity detection and blind docking**.

Molecular docking was p erformed using the CB-Dock2 server, which implements a curvature-based cavity detection algorithm for the identification of potential binding sites. The server identified five primary cavities based on solvent-accessible surface area and topological features. Docking simulations within the identified cavities were performed using AutoDock Vina integrated into the CB-Dock2 pipeline. The transmembrane domain vestibule site (identified as ID 1, volume = 5125 Å^3^) was selected for detailed analysis as it consistently yielded the most favourable binding energyscores and aligned with the known binding pocket of the co-crystallized positive allosteric modulator PNU−120596. The center coordinates (132, 157, 129) and grid dimensions (35 × 21 ×35 Å) were automatically optimized by the server to encompass the inter-subunit interface. Molecular interactions between the ligand atoms and the protein amino acid residues were visualized using BIOVIA Discovery studio (v25.1.0.24284). The docking reliability was assessed by re-docking PNU−120596 into the inter-subunit vestibule of the 7KOX cryo-EM structure. Visual superimposition of the top-ranked docked pose with the experimental co-crystal template revealed a high degree of conformational overlap. Quantitative analysis yielded a maximum common substructure RMSD of 1.036 Å, indicative of good alignment of the core scaffold. Further all-atom comparison confirmed that the ligand maintained the critical spatial orientation required for α7 nAChR allosteric modulation.

### 2.16 Statistical analysis

For behavioral data (neurological score, food buried-seeking, and open field tests), results were presented as mean differences ± SEM with 95% confidence intervals (CI). Pre- and post-treatment comparisons used the CI of the mean difference, whereby a CI excluding zero indicated a significant change; for comparisons between two independent groups, Welch's *t*-test was employed, assuming unequal variances. Other data (odor recognition task, spatial odor memory test, and biochemical data) were presented as mean ± SD and analyzed using one-way ANOVA followed by Tukey's HSD post-hoc test. Hedges' g values were calculated for all applicable comparisons to quantify effect size, interpreted according to the benchmarks proposed by Cohen (1988): g < 0.2, trivial; 0.2–0.5, small; 0.5–0.8, medium; and g > 0.8, large (Cohen, 1998). Statistical significance was set at p < 0.05. All graphs were generated using GraphPad Prism (version 8.01).

## 3 Results

### 3.1 Variation of body temperature during cerebral ischemia induction

**Fig. 3:**
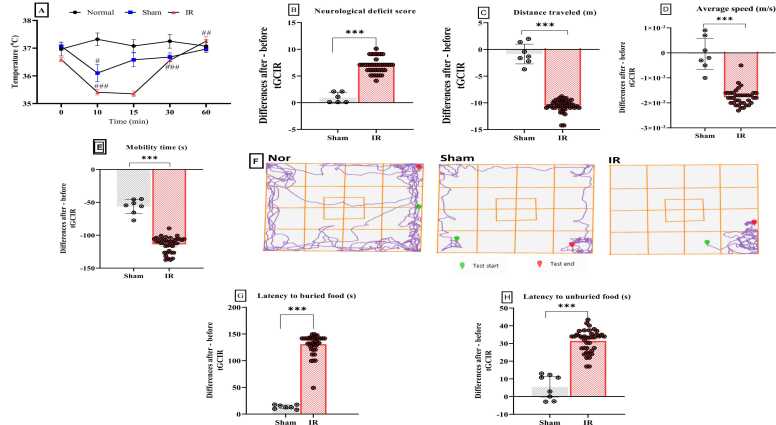
Physiological monitoring and behavioral assessment of cerebral ischemia-reperfusion injury.(A) Core body temperature (°C) recorded at baseline and at 15-minute intervals throughout the surgical procedure in the Normal control (Nor), Sham-operated (Sham), and ischemia-reperfusion (I/R) groups, confirming maintenance of normothermia (36.5–37.5°C) across all groups. (B) Neurological deficit scores assessed at 24 h post-surgery using a standardized 5-point scale. Higher scores indicate greater neurological impairment. (C, D) Total distance traveled (cm) and average velocity (cm/s) recorded during a 5-minute open-field test session, reflecting spontaneous locomotor activity and exploratory behavior. (E) Total mobility time (s) during the open-field test session. (F) Representative movement trajectories of individual rats from each group recorded during the open-field test. Arena dimensions: L: 100 x W: 100 cm x H: 45 cm. Trajectories were generated using ANY-maze 4.8. (G, H) Latency to locate buried food (G) and unburied food (H) in the olfactory food-seeking test (s), used as an index of olfactory-motor coordination. A longer latency indicates greater olfactory or motor impairment. Data are expressed as the mean difference between post- and pre-surgical measurements ± 95% confidence interval (CI). A 95% CI excluding zero was interpreted as evidence of a significant change following the surgical procedure. Group comparisons between I/R and Sham were performed using Welch's *t*-test. ***p < 0.001 vs. Sham group. Nor, n = 7; Sham, n = 7; IR, n = 54. Abbreviations: Nor, normal control group; Sham, sham-operated control group; I/R, transient cerebral ischemia-reperfusion group; CI, confidence interval.

Body temperature dynamics varied significantly across groups during the surgical procedure and observation period. The normal group maintained stability throughout, starting at 36.97 ± 0.25 °C and exhibiting only minor, non-significant fluctuations. In contrast, the surgical groups displayed transient hypothermia, although the severity and recovery patterns differed. The Sham group experienced a significant initial temperature drop of approximately 2.62% (from 37.07 ± 0.15 °C to 36.10 ± 0.31 °C at 10 min, p < 0.05), followed by a gradual non-significant recovery to near baseline 36.97 ± 0.07 °C) by 60 min. The Ischemia-Reperfusion (IR) group experienced the most volatile changes, characterized by a rapid and highly significant initial decrease of approximately 3.00% (from 36.59 ± 0.07 °C to severe hypothermia at 35.41 ± 0.1 °C at 10 min, p < 0.001). This was followed by a sharp and significant recovery, ultimately leading to a potential compensatory hyperthermia (37.25 ± 0.18 °C at 60 min, p < 0.01 vs 30 min). Overall, the IR group demonstrated the most pronounced and dynamic thermal instability, transitioning from severe procedural hypothermia to final hyperthermia (Fig. 3A).

### 3.2 Effect of common and internal carotid arteries occlusion on the neurological score, spontaneous locomotion and exploration in the open arena, and food buried seeking tasks

The assessment of internal and common carotid arteries occlusion revealed a pattern of severe and highly significant neurological and locomotor impairment across multiple measured parameters. Applying a directional hypothesis, neurological deficits (increased score) were significantly elevated in the IR group (6.96 ± 0.22; 95% CI [6.52, 7.40], Hedges’ g = 4.83), while the Sham group showed no significant deficit (0.94 ± 0.40; 95% CI [−0.093, 1.97]). This difference was confirmed as profoundly significant by an independent samples Welch's *t*-test (t (10.00) = 13.16, p < 0.001, Hedges’ g = 4.30) (Fig. 3B). Regarding locomotor activity, both distances traveled (−10.52 ± 0.16 m; 95% CI [−10.84, −10.20], Hedges’ g = 10.07) and average speed (−1.80 × 10^−2^ ± 5.3 × 10^−4^ m/s; 95% CI [−1.90 × 10^−3^, −1.70 × 10^−2^], Hedges’ g =- 4.52) were significantly reduced in the IR group, while the Sham group showed no significant reduction in either metric. Across both distance (t (6.54) = −12.50, p < 0.001, Hedge’s g = −7.66) and speed (t (6.21) = −31.32, p < 0.001, Hedges’ g = −9.49), the impairment observed in the IR group was statistically and substantially greater than in the Sham control (Fig. 3C, D). Notably, mobility time showed a significant reduction in both the Sham group (−56.46 ± 4.38 s; 95% CI [−67.19, −45.74], Hedge’s g = 4.87) and the IR group (−113.8 ± 1.89 s; 95% CI [−117.6, −110.0], Hedges’ g = 9.30); however, the reduction attributable to the occlusion procedure remained highly significant and additive (t(8.39) = −12.013, p < 0.001, Hedges’ g = −4.72) (Fig. 3E). These behavioral alterations were visualized in their movement trajectories within the open field. Indeed, the spatial distribution of locomotor activity within the open field arena revealed distinct patterns across experimental groups. Unoperated control animals exhibited robust and homogeneous exploration, demonstrating a broad and uniform dispersion throughout the maze (Fig. 3F). In contrast, sham-operated animals displayed a discernibly attenuated exploratory behavior, indicating a reduction in overall spatial exploration compared to the unoperated controls (Fig. 3H). Notably, animals subjected to IR exhibited a profound diminution in exploratory activity, with their movements largely confined to a restricted region of the maze, signifying a severe impairment in spatial navigation and exploratory behavior (Fig. 3I).

The analysis of the two food-finding tasks (FBST) assessed the impact of common and internal carotid arteries occlusion on latency, with a positive mean difference indicating significant impairment when the 95% confidence interval (CI) excluded zero. In the buried food task (Fig. 3G), both the Sham group (13.53 ± 1.43 s; 95% CI [10.03, 17.03], Hedge’s g = 3.57) and the IR group (130.9 ± 2.974; 95% CI [124.9, 136.9], Hedge’s g = 6.79) exhibited significant increases in latency post-procedure, indicating that the surgical control procedure itself contributed to the difficulty of the task; however, the impairment in the IR group was statistically and profoundly greater than the Sham group (t(45.51) = 35.563, p < 0.001, Hedge’s g = 6.50). Conversely, in the less complex unburied food task (Fig. 3H), the Sham group did not demonstrate a statistically significant impairment (5.50 ± 2.43 s; 95% CI [−0.25, 11.25]), while the IR group did (31.43 ± 1.01 s; 95% CI [29.38, 33.49], Hedges’ g = 4.79). The difference between groups in this task was also highly significant (t (8.23) = 9.84, p < 0.001, Hedges’ g = 3.96).

### 3.3 Effect of the aqueous extract of *L. camara* on the neurological deficits score

**Fig. 4:**
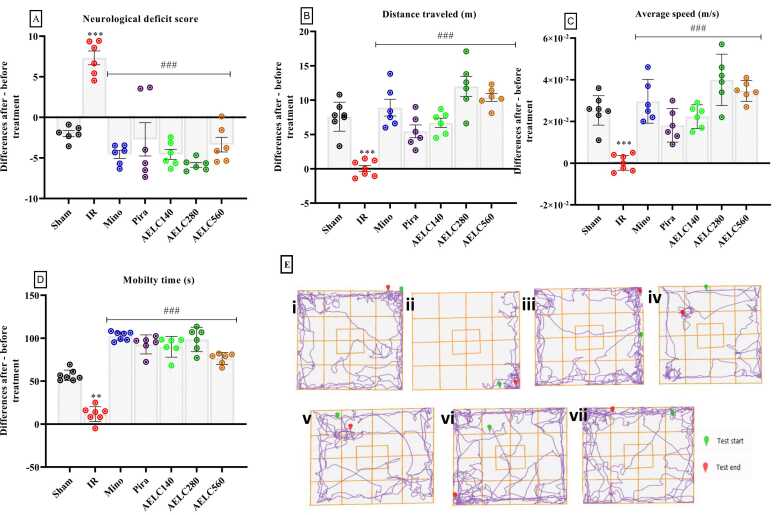
Effect of the aqueous extract of *Lantana camara* on neurological recovery and locomotor behavior following cerebral ischemia-reperfusion injury. (A) Modified Neurological Severity Score (mNSS; scale 0–18) assessed before and after treatment. Lower scores indicate neurological improvement. (B, C) Total distance traveled (cm) and average velocity (cm/s) during a 5-minute open-field test session, reflecting spontaneous locomotor activity. (D) Total mobility time (s) during the open-field test session. (E) Representative movement trajectories of individual rats from each treatment group recorded during the open-field test. Arena dimensions: L: 100 x W: 100 cm x H: 45 cm. Trajectories were generated using ANY-maze. Data are expressed as the mean difference between post- and pre-treatment measurements ± 95% confidence interval (CI). A positive difference with a 95% CI excluding zero was interpreted as evidence of significant improvement following treatment. Data were analyzed by one-way ANOVA followed by Tukey's post-hoc test. ***p < 0.001 vs. Sham (ii); ###p < 0.001 vs. IR (ii). N = 7 per group. Treatment groups: Sham (i), sham-operated rats administered distilled water (10 ml/kg, p.o.); I/R (ii), ischemia-reperfusion rats administered distilled water (10 ml/kg, p.o.); Mino (iii), minocycline (100 mg/kg, p.o.); Pira (iv), piracetam (250 mg/kg, p.o.); AELC−140 (v), aqueous extract of *L. camara* (140 mg/kg, p.o.); AELC−280 (vi), aqueous extract of *L. camara* (280 mg/kg, p.o.); AELC−560 (vii), aqueous extract of *L. camara* (560 mg/kg, p.o.). Abbreviations: AELC, aqueous extract of *Lantana camara*; I/R, transient cerebral ischemia-reperfusion; mNSS, modified Neurological Severity Score; CI, confidence interval; p.o., per os (oral administration).

The IR group demonstrated significant neurological deterioration following the ischemia-reperfusion procedure (7.33 ± 0.86; 95% CI [5.13, 9.52]), with the confidence interval lying entirely above zero, confirming the statistical significance of this deterioration. A Hedges' g value of 3.22 represents a large effect size, indicating that the magnitude of neurological impairment induced by the ischemia-reperfusion procedure was clinically meaningful and substantially beyond chance variation. By contrast, the sham (−2.01 ± 0.38; 95% CI [−2.98, −1.04]), minocycline (−4.57 ± 0.49; 95% CI [−5.81, −3.32]), AELC280 (−5.85 ± 0.29; 95% CI [−6.60, −5.09]), and AELC560 (−3.36 ± 0.90; 95% CI [−5.68, −1.04]) groups all demonstrated statistically significant neurological improvement, with confidence intervals lying entirely below zero. The AELC280 group demonstrated the greatest magnitude of neurological improvement relative to the IR group (Hedges' g = −7.62), followed by minocycline (Hedges' g = −3.53). The piracetam group (−2.71 ± 2.05; 95% CI [−7.98, 2.55]) did not demonstrate statistically significant improvement, since its confidence interval spanned zero. One-way ANOVA confirmed that the IR group exhibited significantly greater neurological severity scores than the sham group (p < 0.001). Relative to the IR group, all treatment groups demonstrated statistically significant improvements in mNSS following Tukey's HSD post hoc analysis (p < 0.001 for all comparisons; Fig. 4A).

### 3.4 Effect of the aqueous extract of *L. camara* on the exploratory and locomotor activity in the open field

Analysis across three key locomotor parameters: distance travelled, average speed, and mobility time, demonstrated that transient cerebral ischemia-reperfusion caused severe locomotor impairment, which was significantly attenuated by all tested treatments (p < 0.001).

Specifically, the vehicle-treated IR group was profoundly impaired, with a mean distance travelled not significantly different from zero (0.06 ± 0.44 m; 95% CI [−1.022, 1.134]) and no significant recovery in average speed (1.90 × 10⁻⁴ ± 1.47 × 10⁻³ m/s; 95% CI [−3.41 × 10⁻³, 3.79 × 10⁻⁴]). By contrast, the sham group and all treatment groups consistently demonstrated statistically significant locomotion and recovery in average speed, with confidence intervals excluding zero in all cases. One-way ANOVA confirmed a significant difference between the sham and IR groups across these parameters (p < 0.001; Fig. 4B and C).

Regarding mobility time, although the IR group demonstrated modest spontaneous recovery (11.63 ± 3.51 s; 95% CI [3.04, 20.22]), this remained significantly lower than that achieved by the sham group (56.79 ± 2.47 s; p < 0.001). All tested treatments: minocycline, piracetam, AELC140, AELC280, and AELC560, resulted in statistically significant restoration of locomotor activity across all three parameters relative to the IR group (p < 0.001 for all comparisons), with the AELC280 group achieving the greatest mean recovery in average speed (4.00 × 10⁻² ± 4.78 × 10⁻³ m/s) and mobility time (98.46 ± 5.56 s), comparable to the reference compound minocycline (102.6 ± 1.92 s). The enhanced locomotor behavior observed in both extract- and minocycline-treated groups was further confirmed by increased exploratory activity in the open field arena, as depicted in Fig. 4E, with recovery levels comparable to those observed in sham animals. All treatments demonstrated large effect sizes across the three locomotor parameters. For distance travelled, AELC140 demonstrated the largest effect size (Hedges' g = 4.52), followed closely by AELC280 and AELC560 (Hedges' g = 4.24 for both). For average speed, the greatest magnitude of recovery was observed with AELC560 (Hedges' g = 7.57). Finally, for mobility time, minocycline exhibited the largest effect size (Hedges' g = 12.15), although AELC extracts and piracetam also demonstrated substantial restoration, with Hedges' g values ranging from 7.05 to 7.87.

.

### 3.5 Effect of the aqueous extract of *L. camara* in the food-buried-seeking test

**Fig. 5:**
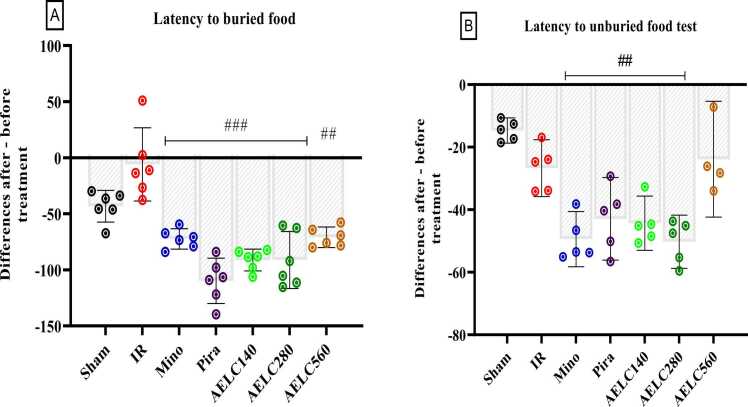
Effect the aqueous extract of *L. camara* on the latency to find the buried (A) and unburied (B) food during the food-buried-seeking test.The data presented shows the mean difference between pre- and post-treatment measurements ± SEM, where *L* =Lower 95% CI of mean and *U* = Upper 95% CI of the mean. N = 7 *per* group. We tested if there was a significant decrease after treatment. If the difference was negative and the 95% confidence interval did not include zero, we concluded a significant improvement. Data were then analyzed by one-way ANOVA, followed by Tukey *post hoc* test. ##*p* < 0.01, ###*p* < 0.001 vs IR. Sham, sham-operated animal treated with distilled water (10 mg/kg, *p.o*.); IR, transient cerebral ischemia-reperfusion group treated with distilled water (10 ml/kg, *p.o*.); AELC (140, 280, 560), *L. camara* at the doses of 140, 280, and 560 mg/kg, respectively *per os*. Mino, Minocycline-treated group (100 mg/kg, *p.o*.); Pira, Piracetam treated group (250 mg/kg, *p.o*.).

Analysis of latency to locate buried food revealed that the vehicle-treated IR group did not demonstrate significant functional restoration (–6.00 ± 31.09 s; 95% CI [–38.63, 26.63]). By contrast, both the sham group (–43.21 ± 13.48 s; 95% CI [–57.36, –29.06]) and all treatment groups demonstrated statistically significant improvement in olfactory detection, with all confidence intervals lying entirely below zero and several treatments exhibiting large within-group effect sizes. The greatest mean improvements were observed in the piracetam group (–109.80 ± 19.25 s; 95% CI [–130.00, –89.61]) and the AELC140 group (–91.24 ± 9.22 s; 95% CI [–100.90, –81.57]).One-way ANOVA confirmed that the sham group demonstrated significantly greater improvement than the IR group (p < 0.001), and all treatment groups demonstrated statistically significant functional restoration relative to the IR group (p < 0.001 for all comparisons). Hedges' g analysis further quantified the magnitude of between-group differences, with the piracetam group demonstrating the largest effect size relative to the IR group (Hedges' g = −3.76), followed by AELC140 (Hedges' g = −3.48), AELC280 (Hedges' g = −2.86), minocycline (Hedges' g = −2.73), and AELC560 (Hedges' g = −2.66; Fig. 5A).With respect to latency to locate unburied food, all groups demonstrated statistically significant improvement following treatment, with all 95% confidence intervals lying entirely below zero. Notably, the vehicle-treated IR group demonstrated substantial spontaneous improvement in locating unburied food (–26.71 ± 7.32 s; 95% CI [–35.80, –17.62]; Hedges' g = −1.99 relative to the sham group), suggesting partial preservation of non-olfactory motivational and motor capacities. Minocycline (–49.42 ± 7.09 s) and AELC280 (–50.24 ± 6.86 s) achieved significantly greater improvement relative to the IR group, as confirmed by large between-group effect sizes (AELC280: Hedges' g = −3.11; minocycline: Hedges' g = −2.95). By contrast, the sham group (–14.70 ± 3.24 s; 95% CI [–18.72, –10.67]) and the AELC560 group (–23.88 ± 11.63 s) demonstrated smaller magnitudes of latency reduction relative to the IR group, with positive Hedges' g values (sham: Hedges' g = 1.99; AELC560: Hedges' g = 0.27) indicating that the IR group paradoxically outperformed these groups on this specific metric, likely reflecting a ceiling effect of spontaneous recovery in the unburied food condition (Fig. 5B).

### 3.6 Effect of the aqueous extract of *L. camara* on the novel odor recognition test

**Fig. 6:**
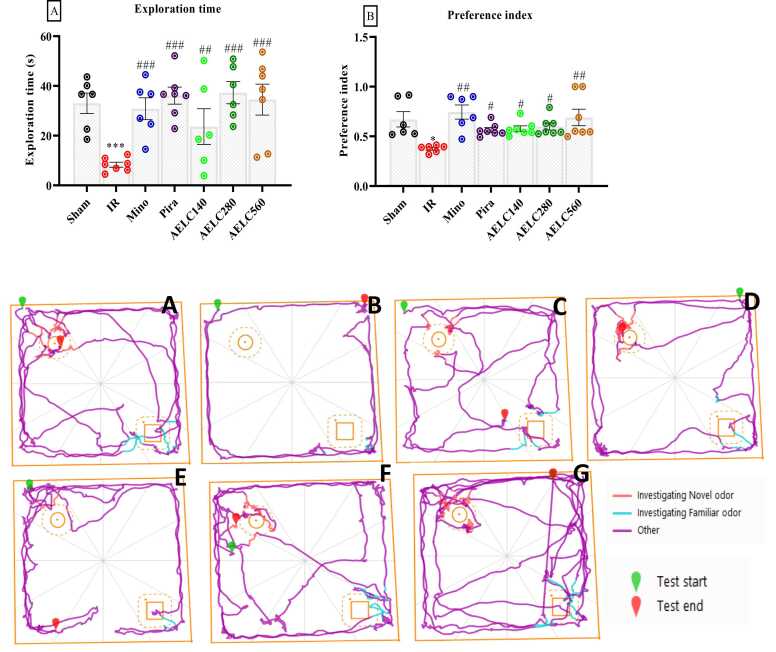
*Upper panel*- Effect of the aqueous extract of *L. camara* on the exploration time (A) and preference index (B) during novel odor recognition test Data are mean ± SEM, N = 7 per group. Data were analyzed by one-way ANOVA, followed by Tukey (HSD). ∗*p* < 0.05, ∗∗∗*p* < 0.001 vs sham; #*p* < 0.05, ##*p* < 0.01, ###*p* < 0.001 vs IR; Sham, sham-operated animal treated with distilled water (10 mg/kg); IR, transient cerebral ischemia-reperfusion group treated with distilled water (10 ml/kg); AELC (140, 280, 560), *L. camara* at the doses of 140, 280, and 560 mg/kg, respectively. Mino, Minocycline- treated group (100 mg/kg); Pira, Piracetam- treated group (250 mg/kg).

Behavioral analysis using the novel odor recognition task provided functional confirmation of the observed neuroprotection, demonstrating a severe olfactory and cognitive deficit following ischemia-reperfusion that significantly impaired the animals' ability to recognize novel odors. This impairment was evidenced by a severely reduced mean exploration time in the IR group (8.28 ± 2.73 s; Hedges' g = −3.19 relative to the sham group) and a marked reduction in the preference index (PI) to 0.38 ± 0.04 (Hedges' g = −2.06 relative to the sham group). Treatment with AELC demonstrated potent neurorestorative effects across both outcome measures. AELC280 produced the greatest restoration of exploration time, representing a 4.50-fold increase relative to the untreated IR group (37.26 ± 10.88 s; p < 0.001; Hedges' g = 3.49). AELC560 achieved the greatest absolute restoration of the PI, representing a 1.82-fold increase relative to the IR group (0.69 ± 0.22; p < 0.01; Hedges' g = 1.88). These treatments successfully reversed the ischaemia-induced deficits, restoring performance to levels at or numerically exceeding sham baseline values (1.13-fold for exploration time and 1.03-fold for PI; Fig. 6, upper panel). Fig. 6, lower panel, illustrates the olfactory investigative behavior of rats towards the odor-emitting objects. Animals in the IR group demonstrated significantly reduced exploratory drive and little to no directed attention towards the odor-emitting objects. By contrast, animals treated with AELC at 280 and 560 mg/kg demonstrated marked novelty-induced exploratory behavior and clear discriminative attention towards the odor-emitting objects, a pattern of behavior comparable to that observed in the sham-operated and minocycline-treated groups (Fig. 6, lower panel).

*Lower panel -* Exploratory trajectories of rats treated with the aqueous extract of the *L. camara* during the novel object recognition test*.* The red plot shows the rat's movement around the novel odor (represented by the circle), while the blue plot shows the movement around the familiar odor (represented by the square). A, sham-operated group; B, transient cerebral ischemia-reperfusion group treated with distilled water (10 ml/kg); C, Minocycline-treated group (100 mg/kg); D, Piracetam-treated group (250 mg/kg); E, F, G, groups treated aqueous extract of *Lantana camara* at the doses of 140, 280, and 560 mg/kg, respectively.

### 3.7 Effect of the aqueous extract of *L. camara* on the spatial odor memory

**Fig. 7:**
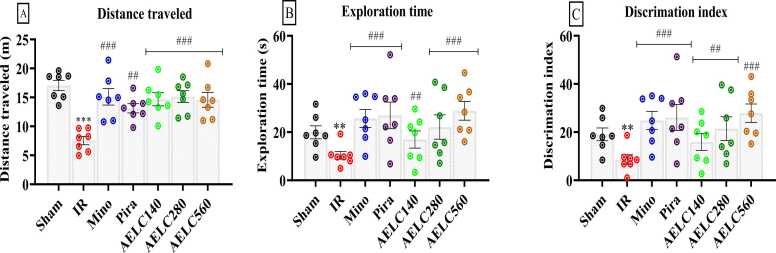
Effect of the aqueous extract of *L. camara* on the distance traveled (A), exploration time (B), and Discriminative index (C) during spatial odor memory test Data are mean ± SEM, N = 7 per group. Data were analyzed by one-way ANOVA, followed by Tukey (HSD). ∗*p* < 0.05, ∗∗*p* < 0.01 vs sham-operated group treated with saline. 0.9%; a*p* < 0.05, b*p* < 0.01, c*p* < 0.001, vs ischemia-reperfusion group treated with distilled water (10 ml/kg); Sham, sham-operated animal treated with distilled water (10 mg/kg); IR, transient cerebral ischemia-reperfusion group treated with distilled water (10 ml/kg) AELC (140, 280, 560), *L. camara* at the doses of 140, 280, and 560 mg/kg, respectively. Mino, Minocycline-treated group (100 mg/kg); Pira, piracetam-treated group (250 mg/kg).

Analysis of the novel odor recognition test demonstrated that the transient cerebral ischemia-reperfusion model induced profound neurocognitive and motor deficits in the animals (Fig. 7). The IR group exhibited severe reductions across all metrics relative to the sham group, notably a 2.26-fold decrease in distance travelled (7.54 ± 1.88 m vs. 17.06 ± 2.33 m; Hedges' g = −4.25; p < 0.001) and a 2.21-fold decrease in the discriminative index (8.64 ± 5.27 vs. 19.08 ± 7.06; Hedges' g = −1.57; p < 0.01). All treatment groups successfully reversed the motor deficit observed in the IR group, with treatments yielding 1.73- to 2.01-fold increases in distance travelled, indicating restored exploratory capacity. The higher extract dose, AELC560 (27.86 ± 10.11; 95% CI [18.50, 37.21]), achieved the largest fold increase in the discriminative index relative to the IR group (3.22-fold; Hedges' g = 2.29; p < 0.001), outperforming the reference compounds minocycline (2.88-fold; Hedges' g = 1.95) and piracetam (3.02-fold; Hedges' g = 1.55). Notably, AELC560 also produced a 3.22-fold increase in exploration time (27.86 ± 10.11 s; 95% CI [19.39, 38.34]; Hedges' g = 2.29; p < 0.001).

### 3.8 Effect of the aqueous extract of *L. camara* on the levels of acetylcholine in the olfactory bulb, piriform cortex, prefrontal cortex, and hippocampus

**Fig. 8:**
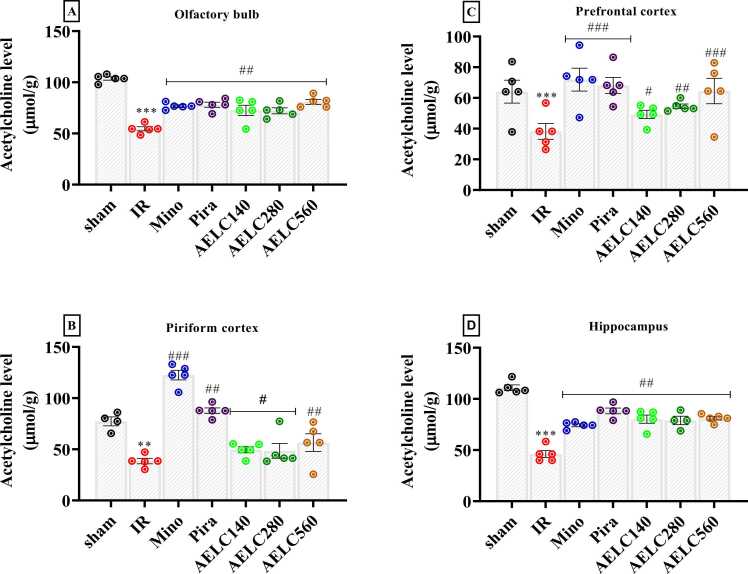
Effect of the aqueous extract of *L. camara* on the levels of acetylcholine in the olfactory bulb (A), piriform cortex (B), prefrontal cortex (C), and hippocampus (D).Data are mean ± SEM, N = 5 per group. Data were analyzed by one-way ANOVA, followed by Tukey (HSD). ∗∗*p* < 0.01, ∗∗∗*p* < 0.001 vs sham; 0.9%; #*p* < 0.05, ##*p* < 0.01, ###*p* < 0.001, vs IR; Sham, sham-operated animal treated with distilled water (10 mg/kg); IR, transient cerebral ischemia-reperfusion group treated with distilled water (10 ml/kg); AELC (140, 280, 560), *L. camara* at the doses of 140, 280, and 560 mg/kg, respectively. Mino, Minocycline- treated group (100 mg/kg); Pira, piracetam- treated group (250 mg/kg).

Acetylcholine (ACh) plays a critical role in mediating olfactory processing and memory consolidation. Analysis demonstrated that ischemia-reperfusion induced severe ACh depletion across all examined brain regions. The most pronounced relative reduction occurred in the hippocampus, where ACh concentrations declined to 41.6% of the sham baseline (IR: 46.08 ± 7.50 μmol/g vs. sham: 110.90 ± 6.29 μmol/g; Hedges' g = −8.46). AELC560 was the most effective dose for restoring cholinergic tone across all examined regions. In the hippocampus, AELC560 (81.03 ± 3.91 μmol/g; 95% CI [76.17, 85.89]) achieved a 1.76-fold restoration relative to the untreated IR group, recovering 73.1% of the sham baseline (Hedges' g = 5.28). ACh levels were similarly restored in the olfactory bulb, where AELC560 (80.91 ± 5.47 μmol/g) produced a 1.48-fold increase relative to the IR group, recovering 78.0% of the sham baseline (Hedges' g = 4.73). A comparable restoration was observed in the piriform cortex, where AELC560 (56.53 ± 19.23 μmol/g) produced a 1.47-fold increase relative to the IR group, recovering 73.1% of the sham baseline (Hedges' g = 1.14). Notably, in the prefrontal cortex, AELC560 (64.49 ± 18.52 μmol/g; 95% CI [41.49, 87.48]) achieved a 1.69-fold increase relative to the IR group, restoring ACh concentrations to sham baseline levels (64.06 ± 16.70 μmol/g; Hedges' g = 1.54). These results demonstrate significant cholinergic restoration by AELC560 across all examined brain regions (p < 0.001 for all AELC560 vs. IR comparisons; Fig. 8).

### 3.9 Effect of the aqueous extract of *L. camara* on nitrites, MDA, and GSH levels

**Fig. 9:**
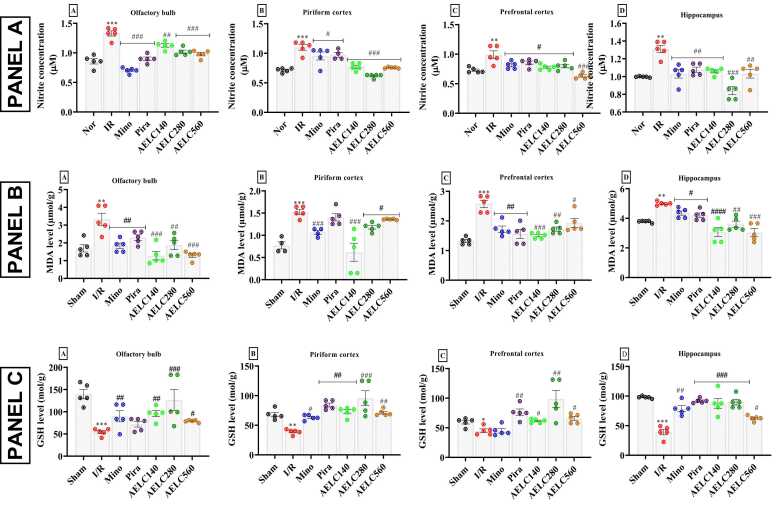
Effect of the aqueous extract of *L. camara* on the level of nitrites (Panel A), MDA (Panel B), and GSH (Panel C) in the olfactory bulb, piriform cortex, prefrontal cortex, and hippocampus Data are mean ± SEM, N = 5 per group. Data were analyzed by one-way ANOVA, followed by Tukey (HSD). ∗*p* < 0.05, ∗∗*p* < 0.01, ∗∗∗*p* < 0.001 vs sham-operated group treated with saline. 0.9%; #*p* < 0.05, ##*p* < 0.01, ###*p* < 0.001, vs ischemia-reperfusion group treated with distilled water (10 ml/kg); Sham, sham-operated animal treated with distilled water (10 mg/kg); IR, transient cerebral ischemia-reperfusion group treated with distilled water (10 ml/kg); AELC (140, 280, 560), *L. camara* at the doses of 140, 280, and 560 mg/kg, respectively. Mino, Minocycline- treated group (100 mg/kg); Pira, Piracetam- treated group (250 mg/kg).

Analysis of nitrite levels revealed that ischemia-reperfusion significantly elevated nitrite concentrations across all four brain regions examined (p < 0.001 for all IR vs. sham comparisons). The greatest IR-induced elevation occurred in the olfactory bulb, with the IR group (1.33 ± 0.04 μM; 95% CI [1.21, 1.45]) demonstrating a 1.55-fold increase relative to the sham group (0.86 ± 0.05 μM; 95% CI [0.74, 0.98]; Hedges' g = 3.76). In this region, AELC560 (0.98 ± 0.03 μM; 95% CI [0.90, 1.06]) was most effective, reducing nitrite levels by a factor of 0.74 relative to the IR group (Hedges' g = 4.24; p < 0.001). The most pronounced reduction was observed in the piriform cortex, which also exhibited a substantial IR-induced elevation (1.54-fold): AELC280 (0.62 ± 0.02 μM; 95% CI [0.58, 0.66]) produced the largest effect size (Hedges' g = 3.10) and greatest relative reduction (0.56-fold relative to the IR group; 1.10 ± 0.05 μM; 95% CI [0.97, 1.23]; p < 0.01), yielding a final nitrite level below the sham baseline (0.72 ± 0.02 μM). In the hippocampus, despite the lowest IR-induced fold-increase (1.31-fold), the effect of AELC treatment remained substantial. AELC280 (0.84 ± 0.05 μM; 95% CI [0.71, 0.96]) achieved a 0.64-fold reduction relative to the IR group (1.31 ± 0.04 μM; 95% CI [1.20, 1.43]; p < 0.01), effectively restoring nitrite levels to 84.2% of the sham value (1.00 ± 0.01 μM). Finally, in the prefrontal cortex, IR caused a 1.36-fold increase in nitrite concentration (0.99 ± 0.07 μM; 95% CI [0.81, 1.17]), with AELC560 (0.63 ± 0.02 μM; 95% CI [0.58, 0.69]) providing the most effective protection, achieving a 0.64-fold reduction (Hedges' g = 3.35; p < 0.001) and a final nitrite level below that of the sham group (0.73 ± 0.02 μM; Fig. 9, panel A).

Analysis of malondialdehyde (MDA) levels revealed a consistent elevation following ischemia-reperfusion and significant attenuation by AELC across all examined regions. The olfactory bulb exhibited the highest IR-induced MDA concentration, with the IR group (3.33 ± 0.34 μmol/g; 95% CI [2.38, 4.27]) demonstrating a 1.97-fold increase relative to the sham group (1.69 ± 0.20 μmol/g; 95% CI [1.12, 2.26]; Hedges' g = 2.61). AELC140 (1.28 ± 0.24 μmol/g; 95% CI [0.62, 1.93]) produced a substantial 0.38-fold reduction relative to the IR group (Hedges' g = 3.12), reducing MDA concentrations below the sham baseline. The piriform cortex similarly exhibited a near 2-fold MDA elevation in the IR group (1.52 ± 0.06 μmol/g; 95% CI [1.37, 1.68]) relative to the sham group (0.77 ± 0.08 μmol/g), with AELC140 (0.62 ± 0.20 μmol/g; 95% CI [0.06, 1.18]) again demonstrating the greatest efficacy, reducing MDA levels by a factor of 0.41 (Hedges' g = 2.72) and yielding a final concentration significantly below the sham baseline. In the prefrontal cortex, the IR group (2.58 ± 0.12 μmol/g; 95% CI [2.23, 2.92]) exhibited a 1.96-fold elevation relative to the sham group (1.32 ± 0.05 μmol/g), with AELC producing the largest effect size across all examined regions (Hedges' g = 5.93). AELC140 (1.50 ± 0.04 μmol/g; 95% CI [1.39, 1.62]) produced a 0.58-fold reduction in MDA concentration (Hedges' g = 5.24). Finally, the hippocampus exhibited the highest absolute MDA concentration in the IR group (5.00 ± 0.06 μmol/g; 95% CI [4.84, 5.15]), though the fold increase relative to sham was the lowest across all regions (1.32-fold). AELC560 (3.04 ± 0.26 μmol/g; 95% CI [2.32, 3.77]) produced the most effective reduction, achieving a 0.61-fold decrease relative to the IR group (Hedges' g = 4.63; p < 0.001) and restoring MDA concentrations to approximately 80% of the sham baseline (3.78 ± 0.03 μmol/g; Fig. 9, panel B).

Analysis of glutathione (GSH) levels confirmed that ischemia-reperfusion significantly depleted GSH reserves across all examined brain regions. The most severe GSH depletion occurred in the hippocampus, where the IR group (39.2 ± 4.60 μmol/g; 95% CI [26.4, 52.0]) exhibited a reduction to 0.40 of the sham value (97.9 ± 0.87 μmol/g; 95% CI [95.5, 100.0]; Hedges' g = 7.92; p < 0.001). Treatment with AELC280 (89.8 ± 4.87 μmol/g; 95% CI [76.3, 103.0]) produced a 2.29-fold restoration relative to the IR group (Hedges' g = 4.77; p < 0.01), restoring GSH to 91.7% of the sham baseline. A comparably strong effect was observed in the olfactory bulb, where the IR group (54.2 ± 3.56 μmol/g; 95% CI [44.4, 64.1]) exhibited a 0.39-fold depletion relative to sham (Hedges' g = 4.95). AELC280 (127.0 ± 23.80 μmol/g; 95% CI [60.61, 193.01]) restored GSH by a factor of 2.34 relative to the IR group (Hedges' g = 1.91; p < 0.01), reaching 90.7% of the sham baseline. In the piriform cortex, AELC280 (95.71 ± 12.50 μmol/g; 95% CI [61.00, 130.00]) achieved the greatest relative restoration, increasing GSH by 2.45-fold relative to the IR group (39.01 ± 1.96 μmol/g; 95% CI [33.60, 44.40]; Hedges' g = 2.83; p < 0.01), yielding GSH levels exceeding the sham baseline (68.12 ± 3.74 μmol/g) by 1.41-fold. Finally, in the prefrontal cortex, the IR group demonstrated the least severe GSH depletion across all regions, with concentrations reduced to 0.76 of the sham value (Hedges' g = 2.08). Nevertheless, AELC280 (98.71 ± 14.40 μmol/g; 95% CI [58.90, 138.50]) produced the largest absolute GSH increase across all regions, representing a 2.19-fold increase relative to the IR group (45.00 ± 3.11 μmol/g; 95% CI [36.40, 53.60]; Hedges' g = 2.31; p < 0.01) and a final GSH concentration 1.66-fold above the sham baseline (59.51 ± 3.13 μmol/g; Fig. 9, panel C).

### 3.10 Effect of the aqueous extract of *L. camara* on TNF-α and IL-1β levels

**Fig. 10:**
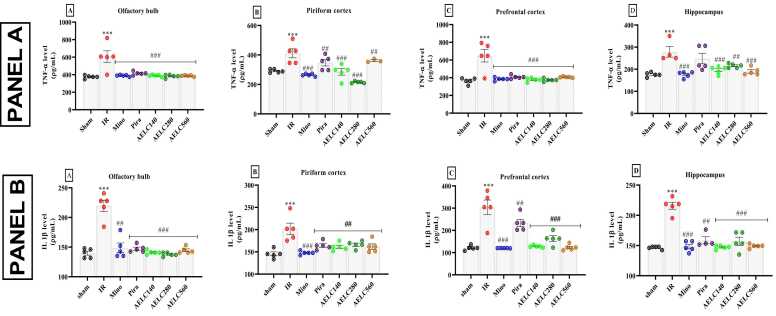
Effect of the aqueous extract of *L. camara* on the levels of TNF-α and Interleukin 1 beta in the olfactory bulb, piriform cortex, prefrontal cortex, and hippocampus.Data are mean ± SEM, N = 5 *per* group. Data were analyzed by one-way ANOVA, followed by Tukey (HSD). ∗*p* < 0.05, ∗∗*p* < 0.01, ∗∗∗*p* < 0.001 vs sham-operated group treated with saline. 0.9%; #*p* < 0.05, ##*p* < 0.01, ###*p* < 0.001, vs ischemia-reperfusion group treated with distilled water (10 ml/kg); Sham, sham-operated animal treated with distilled water (10 mg/kg); IR, transient cerebral ischemia-reperfusion group treated with distilled water (10 ml/kg); AELC (140, 280, 560), *L. camara* at the doses of 140, 280, and 560 mg/kg, respectively. Mino, Minocycline- treated group (100 mg/kg); Pira, Piracetam- treated group (250 mg/kg).

Cytokine analysis revealed that cerebral ischemia-reperfusion significantly elevated TNF-α levels across all examined brain regions (p < 0.001), with the greatest fold increase observed in the prefrontal cortex, where the IR group (648.20 ± 70.18 pg/ml; 95% CI [453.40, 843.10]) exhibited a 1.78-fold elevation relative to the sham group (364.51 ± 14.81 pg/ml; 95% CI [323.41, 405.62]; Hedges' g = 2.50). AELC demonstrated significant anti-inflammatory effects across all examined regions. In the prefrontal cortex, AELC280 (374.9 ± 7.41 pg/ml; 95% CI [354.31, 395.42]) was the most effective dose, achieving a 0.58-fold reduction relative to the IR group (Hedges' g = 2.45; p < 0.01). In the olfactory bulb, AELC280 (383.0 ± 8.33 pg/ml; 95% CI [359.93, 406.24]) achieved a 0.63-fold reduction in TNF-α relative to the IR group (606.73 ± 66.42 pg/ml; 95% CI [422.30, 791.20]). The most pronounced effect was observed in the piriform cortex, where AELC280 (214.71 ± 2.96 pg/ml; 95% CI [206.50, 223.00]) produced a 0.52-fold reduction relative to the IR group (411.22 ± 30.69 pg/ml; 95% CI [326.01, 496.43]; Hedges' g = 4.03; p < 0.01), reducing TNF-α concentrations below the sham baseline (291.30 ± 5.29 pg/ml; 95% CI [276.72, 306.02]). Finally, in the hippocampus, AELC560 (190.0 ± 7.52 pg/ml; 95% CI [169.13, 210.94]) was the most effective dose, achieving a 0.68-fold reduction relative to the IR group (278.82 ± 24.09 pg/ml; 95% CI [202.11, 355.54]; p < 0.001; Fig. 10, panel A). Cytokine analysis for IL-1β revealed that ischemia-reperfusion induced significant neuroinflammatory responses across all examined brain regions (p < 0.001), with the most severe elevation observed in the prefrontal cortex, where the IR group (304.31 ± 32.28 pg/ml; 95% CI [214.62, 393.92]) demonstrated a 2.51-fold increase relative to the sham group (121.22 ± 4.65 pg/ml; 95% CI [108.33, 134.13]; Hedges' g = 3.55). AELC demonstrated potent anti-inflammatory effects, particularly in normalizing IL-1β concentrations towards sham levels. In the prefrontal cortex, AELC560 (123.12 ± 5.87 pg/ml; 95% CI [106.82, 139.42]) achieved the greatest reduction across all regions, producing a 0.41-fold decrease relative to the IR group (Hedges' g = 3.49; p < 0.001). Near-complete normalization was also observed in the olfactory bulb, where the IR group (219.74 ± 9.57 pg/ml; 95% CI [193.10, 246.20]) exhibited a 1.57-fold elevation relative to sham: AELC280 (137.7 ± 1.36 pg/ml; 95% CI [133.90, 141.50]) produced a 0.63-fold decrease (Hedges' g = 5.37; p < 0.01). In the hippocampus, the IR group (215.93 ± 5.88 pg/ml; 95% CI [199.60, 232.20]) exhibited a comparable inflammatory elevation, with AELC140 (147.31 ± 1.34 pg/ml; 95% CI [143.60, 151.00]) producing a 0.68-fold reduction that restored IL-1β concentrations to near-sham levels (146.54 ± 1.06 pg/ml; Hedges' g = 7.19). By contrast, the piriform cortex exhibited the lowest IR-induced IL-1β elevation (1.39-fold), and the most effective dose, AELC140 (161.2 ± 3.91 pg/ml; 95% CI [150.40, 172.10]), produced a comparatively modest 0.80-fold reduction (Hedges' g = 1.94; Fig. 10, panel B).

### 3.11 Effect of the aqueous extract of *L. camara* on the histology of the olfactory bulb, piriform cortex, prefrontal cortex, and hippocampus

**Fig. 11:**
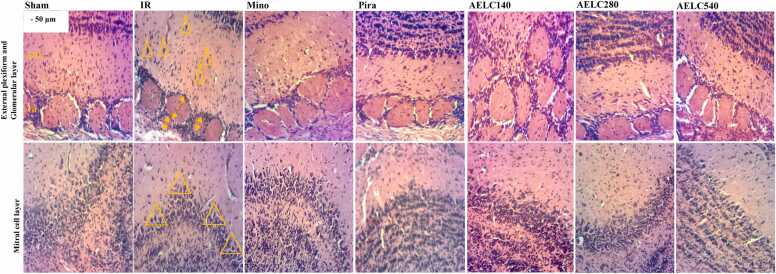
Microphotographs of the different layers of the olfactory bulb after hematoxylin-eosin stain (× 250). EPL, external plexiform layer; GL, Glomerular layer; sham, sham-operated group; IR, transient global ischemia/reperfusion; Mino, minocycline-treated group (100 mg/kg); Pira, piracetam-treated group (250 mg/kg); AELC (140, 280, 560), aqueous extract of *L. camara* (at doses of 140, 280, 560 mg/kg, respectively). Normal neurons can be obtained with rounded nuclei and intact nucleoli. Hypertrophic cells with hyperchromatic nuclei (triangles), and diameter of glomeruli (double arrow head line) are characteristics of injured olfactory bulb. Scale bar −50 µm.

The olfactory bulb plays a critical role in odorant detection, with its constituent layers serving distinct functional roles: the glomerular layer mediates the reception and organization of sensory inputs and contributes to odor sensitivity and discrimination; the external plexiform layer modulates and refines olfactory information; and the mitral cell layer integrates input from the glomeruli and relays it to downstream brain areas. Hematoxylin and eosin-stained sections of the olfactory bulb in the IR group revealed marked disorganization of the external plexiform, glomerular, and mitral cell layers (Fig. 11), with semi-quantitative scoring confirming moderate neuronal loss (score 2; κ = 0.71) and mild-to-moderate ghost cell formation (score 2; κ = 0.67). Additional pathological features included shrunken and pyknotic nuclei, erythrocytic infiltration, astrogliosis, and glomerular dystrophy. Mild neuropil vacuolation (score 1; κ = 0.72) and minor tissue disruption (score 1; κ = 0.82) were also recorded. Treatment with AELC at all doses substantially attenuated these alterations, reducing neuronal density and ghost cell scores to 1 and tissue integrity scores to 0 at the 280 and 560 mg/kg doses, consistent with the effects of minocycline and piracetam. These improvements were absent or minimal in the sham control group, which returned scores of 0 across all criteria.

**Fig. 12:**
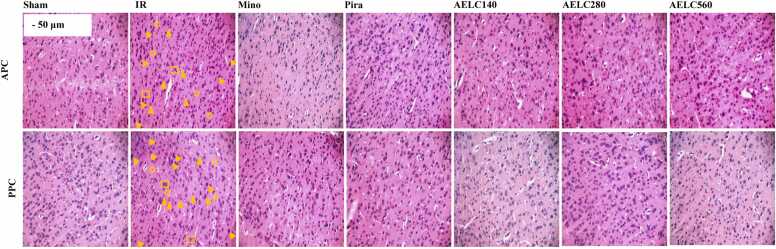
*M*icrophotographs of the anterior and posterior piriform cortices after hematoxylin-eosin stain (× 250). APC, anterior piriform cortex; PFC, posterior piriform cortex; sham, sham-operated group; IR, transient global ischemia/reperfusion; Mino, minocycline-treated group (100 mg/kg); Pira, piracetam-treated group (250 mg/kg); AELC (140, 280, 560), aqueous extract of *L. camara* (at doses of 140, 280, 560 mg/kg, respectively). Normal neurons (boxes) can be obtained with rounded nuclei and intact nucleoli. Prominent eosinophilic cytoplasm with or without shrunken nuclei (arrowhead), pyknotic nuclei (full arrows), and neuron swelling and vacuolation (circles) are characteristics of degenerating neurons. Scale bar −50 µm.

The anterior piriform cortex is implicated in odor detection, while the posterior piriform cortex contributes to odor processing and olfactory memory. Hematoxylin and eosin staining of both piriform cortex subdivisions (Fig. 12) revealed several histopathological abnormalities in the IR group, including prominent cytoplasmic eosinophilia, karyopyknosis, neuronal swelling, and vacuolation, corroborated by scores of 1 for neuronal density (κ = 0.63), 2 for ghost cell formation (κ = 0.77), and 2 for both neuropil vacuolation (κ = 0.76) and tissue integrity (κ = 0.83). These alterations were significantly more pronounced in untreated IR animals relative to both sham-operated controls and extract-treated groups. AELC at all doses reduced all four criteria scores to 0 or 1 in the piriform cortex, with AELC280 achieving scores of 0 for neuronal density and tissue integrity (κ = 0.72 – 0.87), comparable to the reference compounds.

**Fig. 13:**
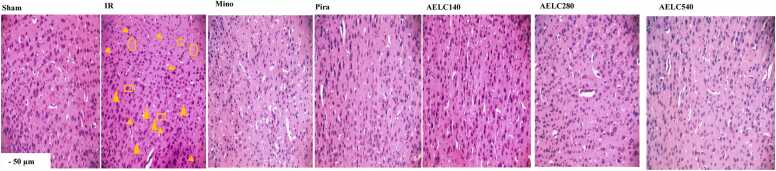
*M*icrophotographs of the prefrontal cortex after hematoxylin-eosin stain (× 250). Sham, sham-operated group; IR, transient global ischemia/reperfusion; Mino, minocycline-treated group (100 mg/kg); Pira, piracetam-treated group (250 mg/kg); AELC (140, 280, 560), aqueous extract of *L. camara* (at doses of 140, 280, 560 mg/kg, respectively). Normal neurons (boxes) can be obtained with rounded nuclei and intact nucleoli. Prominent eosinophilic cytoplasm with or without shrunken nuclei (arrowhead), pyknotic nuclei (full arrows), and neuron swelling and vacuolation (circles) are characteristics of degenerating neurons. Scale bar −50 µm.

Hematoxylin and eosin-stained sections of the prefrontal cortex (Fig. 13) similarly demonstrated numerous pathological features in IR animals, including prominent eosinophilic cytoplasm with or without nuclear shrinkage, pyknotic nuclei (solid arrows), and neuronal swelling and vacuolation (circles), corresponding to semi-quantitative scores of 1 for neuronal density (κ = 0.71), ghost cell formation (κ = 0.62), neuropil vacuolation (κ = 0.79), and tissue integrity (κ = 0.72). AELC280 and AELC560 restored neuronal density, tissue integrity, and vacuolation scores to 0 in this region (κ = 0.67–0.92), with the highest interrater agreement recorded for the prefrontal cortex across all criteria (κ up to 0.92), indicating almost perfect concordance between raters (Table 2).

**Fig. 14:**
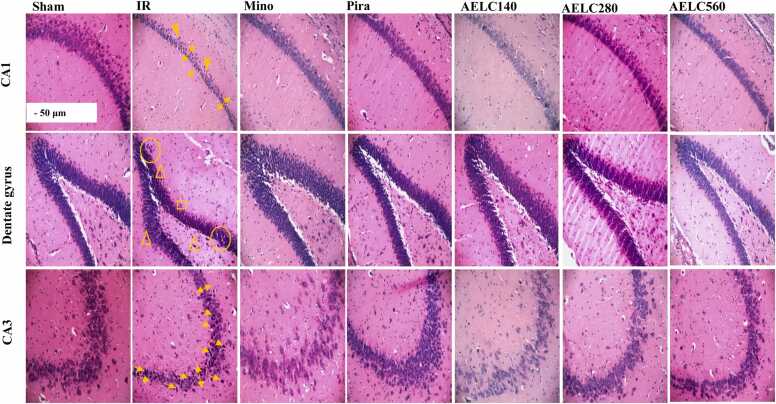
*M*icrophotographs of the *Cornu ammonis* 1, dentate gyrus, and *Cornu ammonis* 3 regions of the hippocampus after hematoxylin-eosin stain (× 250). CA, *Cornu ammonis*; Sham, sham-operated group; IR, transient global ischemia/reperfusion; Mino, minocycline-treated group (100 mg/kg); Pira, piracetam-treated group (250 mg/kg); AELC (140, 280, 560), aqueous extract of *L. camara* (at doses of 140, 280, 560 mg/kg, respectively). Normal neurons (boxes) can be obtained with rounded nuclei and intact nucleoli. Prominent eosinophilic cytoplasm with or without shrunken nuclei (arrowhead), astrogliosis (full arrows and rectangles), and granule cell disorganization (circles) are characteristics of degenerating neurons. Scale bar −50 µm.

Hematoxylin and eosin staining of hippocampal sections (Fig. 14) revealed the most severe pathological burden among all examined regions in untreated IR animals. Extensive neuronal loss and disorganization were observed in the granule cell layer of the dentate gyrus hilus, accompanied by astrogliosis characterized by hypertrophic cells with prominent nuclei. The CA1 and CA3 subfields similarly exhibited marked pathological changes, including reduced neuronal diameter and evidence of neurodegeneration. Semi-quantitative scoring confirmed moderate neuronal loss (score 2; κ = 0.64), the highest ghost cell burden across all regions (score 3; κ = 0.77), moderate neuropil vacuolation (score 2; κ = 0.76), and clear tissue architectural disruption (score 2; κ = 0.83). AELC280 and AELC560 restored neuronal density and tissue integrity scores to 0 in the hippocampus (κ = 0.76–0.83), with interrater reliability ranging from substantial to almost perfect across all criteria (κ = 0.64–0.83), lending methodological credibility to these findings (Table 2).

Across all four brain regions and histological criteria, a consistent dose-dependent pattern was observed: IR induced the highest pathological scores, which were progressively attenuated with increasing AELC doses, with AELC280 and AELC560 achieving near-normalization of all scores to 0 or 1, comparable to the neuroprotective effects of minocycline (100 mg/kg) and piracetam (250 mg/kg). Interrater reliability was uniformly substantial to almost perfect across all groups and criteria (κ = 0.60–0.92), with the sole exception of one AELC560 ghost cell assessment in the olfactory bulb (κ = 0.60), confirming the methodological robustness of the scoring procedure (Table 2).

### 3.12 Compounds identified from the extract of *L. camara*

**Fig. 15:**
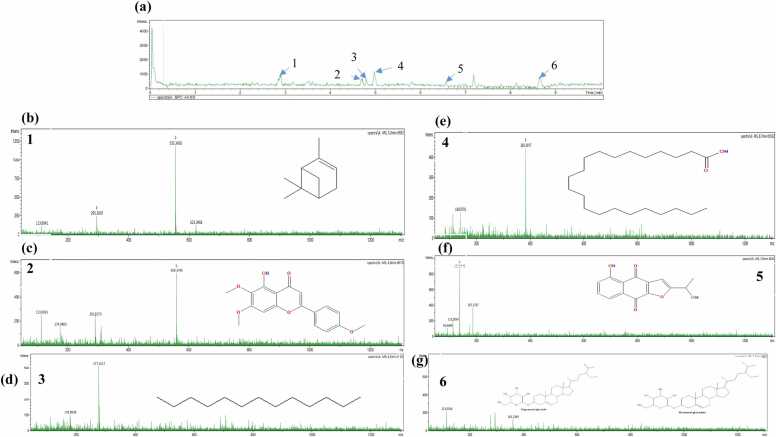
Total ion chromatogram of the aqueous extract of *Lantana camara* (a) and mass spectra of individual compounds detected (b to g). 1 – 6: Compound ID.

Fig. 15 presents the total ion chromatogram obtained from LC-MS analysis of the *Lantana camara* aqueous extract. The chromatogram displays ion intensity as a function of retention time, with each observed peak representing an individual phytoconstituent separated from the crude extract. Six prominent chromatographic peaks were putatively identified, corresponding to six phytoconstituents present in the *L. camara* aqueous extract. Table 1 catalogues these identified compounds, listed in ascending order of chromatographic retention time.

**Table 1:** List of phyto-active compounds identified from the AELC.

**Compound ID**	**Name**	**Retention time (min)**	**Chemical class**	**Molecular formula**	**Exact*****m/z***	**Precursor ion**	**Mean relative abundance (%) ± SEM**
1	Alpha-pinene	2.9 **±** 0.1	Bicyclic monoterpenoid	C_10_H_16_	136.23	M + H	5.23 ± 0.87
2	Salvigenin	4.7 **±** 0.1	Flavonoid	*C*_18_*H*_16_*O*_6_	328.3	M + H	3.43 ± 0.32
3	Behenic acid	4.8 **±** 0.1	Saturated fatty acid	C_21_H_43_COOH	340.6	[M]^–^	2.62 ± 0.34
4	Tridecane	5.0 **±** 0.1	Alkane	C_13_H_28_	184.36	M-H	4.62 ± 0.54
5	Kigelinone	6.6 **±** 0.1	**Naphthoquinones**	C_14_H_10_O_5_	239.2	M -H2O-H	2.82 ± 0.30
6	Charantin	8.7 **±** 0.1	Steroid glycosides	C_35_H_60_O_6_ (*β*-sitosteryl glucoside)	1151.7	2M-H	
				C_35_H_58_O_6_ (5.22-stigmasteryl glucoside)			4.32 ± 0.14

*m/z*: Mass-to-charge ratio, *SEM:* standard error on mean; *N* = 3.

**Table 2:** Effect of the extract on the microarchitecture of the olfactory bulb, piriform cortex, prefrontal cortex, and hippocampus.

### 3.13 Drug-likeness, membrane permeance, and predicted toxicity of phytoactive constituents of *Lantana camara* aqueous extract

**Fig. 16:**
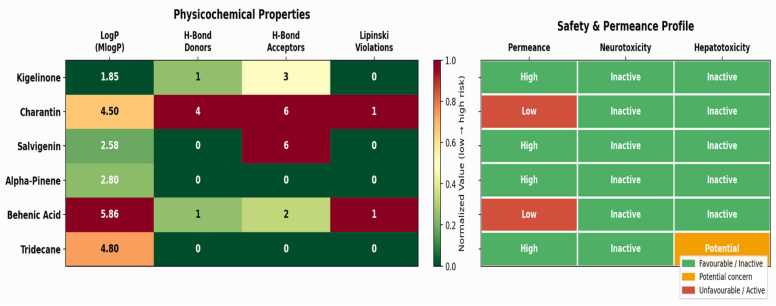
Drug-likeness, membrane permeance, and predicted toxicity of phytoactive constituents of *Lantana camara* aqueous extract.LogP (MlogP): Octanol-Water Partition Coefficient, measuring lipophilicity. H-Bond Donors: number of NH or OH groups. H-Bond Acceptors: number of N or O atoms with lone pairs. Lipinski Violations: number of Lipinski Rule of Five criteria not met (MW > 500 Da, LogP > 4.15, H-bond donors > 5, H-bond acceptors > 10). Permeance reflects predicted membrane penetration capacity. Neurotoxicity and Hepatotoxicity predictions derived from ProTox−3.0. .

*In silico* profiling of the six phytoactive compounds identified in AELC was conducted using Lipinski's Rule of Five and ProTox-3.0 toxicity predictions to assess their pharmacokinetic suitability and preliminary safety profiles (Fig. 16). Four compounds: tridecane, alpha-pinene, Salvigenin, and Kigelinone, satisfied all four Lipinski criteria with zero violations, indicating favorable predicted oral bioavailability. The remaining two compounds each recorded a single violation: behenic acid exceeded the lipophilicity threshold (LogP = 5.86 > 4.15), and Charantin exceeded the molecular weight threshold (MW > 500 Da), consistent with its bulky steroid glycoside architecture. Across the dataset, LogP values ranged from 1.85 (Kigelinone) to 5.86 (behenic acid), with Kigelinone and Salvigenin (LogP = 2.58) presenting the most balanced lipophilicity profiles within the optimal range for simultaneous membrane permeation and aqueous solubility. Hydrogen bond donor counts ranged from 0 to 4, with Charantin recording the highest value (4), while hydrogen bond acceptor counts ranged from 0 to 6, with Salvigenin and Charantin sharing the highest values (6 each).

Membrane permeance predictions were consistent with the physicochemical profiles. High permeance was predicted for tridecane, alpha-pinene, Salvigenin, and Kigelinone, attributed respectively to high lipophilicity, small non-polar structure, high gastrointestinal absorption, and favorable overall physicochemical balance. Low permeance was predicted for behenic acid, owing to poor aqueous solubility consequent upon its excessive lipophilicity, and for Charantin, reflecting its large molecular bulk and steroid glycoside structure. Toxicity predictions were broadly favorable across the dataset. Five of the six compounds: tridecane, behenic acid, alpha-pinene, Salvigenin, and Charantin, were predicted to be inactive for both neurotoxicity and hepatotoxicity, supporting their preliminary safety for neurological applications. The sole exception was Kigelinone, which, despite recording zero Lipinski violations, the lowest LogP in the dataset (1.85), and high predicted membrane permeance, was flagged for potential hepatotoxicity by ProTox−3.0 prediction. No compound in the dataset was predicted to be active for neurotoxicity.

Integrating drug-likeness, permeance, and toxicity data, Salvigenin and Kigelinone emerged as the most pharmacologically promising lead compounds. Both satisfied all Lipinski criteria, exhibited high predicted membrane permeance, and demonstrated favorable neurotoxicity profiles. Kigelinone's optimal LogP and zero violations position it as the most balanced candidate for blood-brain barrier penetration and central nervous system bioavailability, pending resolution of its predicted hepatotoxicity risk through future *in vivo* hepatic safety assessments. Salvigenin's clean dual toxicity profile and high gastrointestinal absorption further support its candidacy as a neuroprotective scaffold warranting targeted isolation and functional validation (Fig. 16).

### 3.14 In *silico* identification of the α7 nAChR transmembrane vestibule as a high-affinity target for *L. camara* phytochemicals

**Fig. 17:**
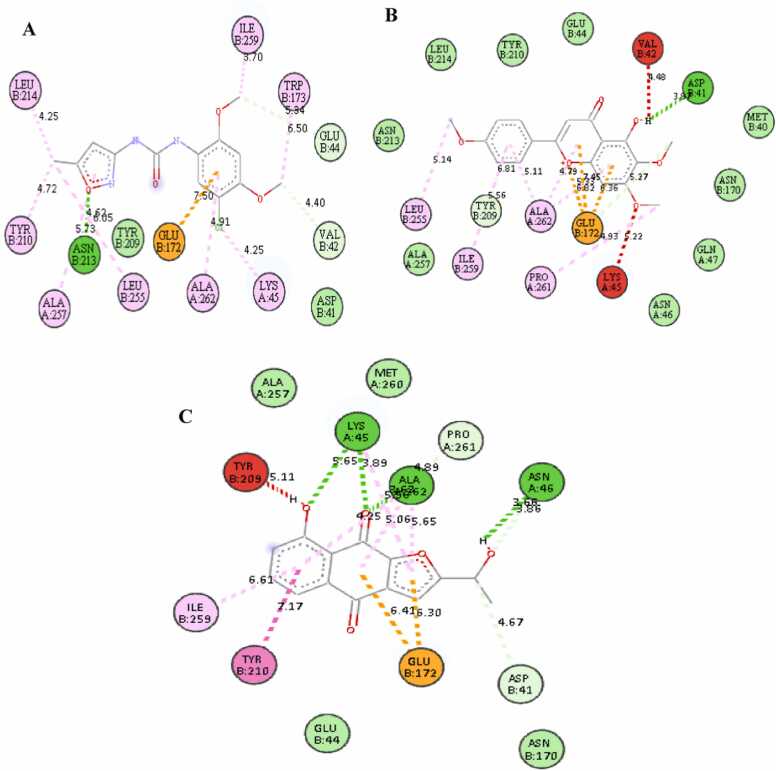
Two-dimensional molecular interaction maps of ligands within the α7 nAChR intersubunit allosteric pocket. The diagrams illustrate the binding poses and intermolecular forces for (A) PNU−120596 (reference modulator), (B) Salviginin, and (C) Kigelinone. Interaction types are color-coded: conventional hydrogen bonds (green), π-anion and π- π interactions (orange/purple), and hydrophobic alkyl/ π -alkyl contacts (pink). Note the high-strength hydrogen bond between Kigelinone and Ala262 and the unfavorable steric/electrostatic interaction (red) between Salviginin and Lys45. Residues from Chain A and Chain B are labeled to indicate the intersubunit nature of the binding site.

To investigate the binding potential of *L. camara* phytochemicals without a priori bias, blind docking was performed using the CB-Dock2 server. Of the five cavities detected, the transmembrane domain (TMD) vestibule (volume = 5125 Å³) emerged as the primary consensus binding site for all tested ligands. Comparative docking analysis revealed that kigelinone demonstrated greater predicted binding affinity (AutoDock Vina score: −7.5 kcal/mol) relative to the reference modulator PNU−120596 (−7.3 kcal/mol; Table 3). This favorable binding score was underpinned by a dense network of short-range hydrogen bonds within the inter-subunit interface. Most notably, Kigelinone formed a high-strength hydrogen bond with Ala262 (chain A) at a distance of 2.67 Å, complemented by stabilizing contacts with Lys45 (chain A) and Asn46 (chain A). These interactions bridged the interface between chain A and chain B, a binding mode further reinforced by π-π T-shaped stacking with Tyr210 (chain B) at 7.17 Å (Fig. 17).

**Table 3:** Molecular docking parameters and interacting residues of *Lantana camara* ligands with the α7 nAChR (PDB ID: 7KOX).

**Ligands**	**Vina score (kcal/mol)**	**Cavity volume (**Å^3^**)**	**Grid center (x,y,z)**	**Key interacting residues**
**PNU−120596 (*****control*****)**	−7.3	5125	132, 157, 129	**Ch A:** GLU44, LYS45, ASN46, GLN47, SER126, CYS127, TYR128, CYS141, LYS142, LEU254, ALA257, GLU258, MET260, PRO261, ALA262, THR263 **Ch B:** LEU37, GLN38, ILE39, MET40, ASP41, VAL42, ASP43, GLU44, LYS45, ILE168, ASN170, GLU172, TRP173, ARG205, TYR209, TYR210, ASN213, LEU214, LEU255, GLU258, ILE259, MET260
**Salviginin**	−7.2	5125	132, 157, 129	**Ch A:** LYS45, ASN46, GLN47, ASN93, SER126, CYS127, TYR128, ILE129, HIS140, CYS141, LYS142, LEU254, ALA257, GLU258, MET260, PRO261, ALA262, THR263 **Ch B:** LEU37, GLN38, ILE39, MET40, ASP41, VAL42, ASP43, GLU44, LYS45, TRP54, TYR167, ILE168, ASN170, GLU172, TRP173, ARG205, TYR209, TYR210, ASN213, LEU214, VAL251, LEU254, LEU255, GLU258, ILE259
**Kigelinone**	−7.5	5125	132, 157, 129	**Ch A:** LYS45, ASN46, GLN47, TYR92, ASN93, SER126, CYS127, TYR128, HIS140, CYS141, LYS142, LYS144, LEU254, LEU255, ALA257, GLU258, MET260, PRO261, ALA262 **Ch B:** LEU37, MET40, ASP41, VAL42, ASP43, GLU44, TRP54, GLY166, ILE168, ASN170, GLU172, TRP173, ARG205, TYR209, TYR210, ASN213, LEU214, LEU254, LEU255, GLU258, ILE259

By contrast, while PNU−120596 and Salvigenin utilized Glu172 (chain B) as a common π-anion anchor, their binding profiles were characterized by considerably longer interaction distances (up to 7.50 Å for PNU−120596), suggesting a more diffuse binding mode. The predicted binding affinity of Salvigenin (−7.2 kcal/mol) was potentially constrained by a relatively long hydrogen bond with Asp41 (chain B) at 3.87 Å and an unfavorable interaction with Lys45 (chain A) at 5.22 Å, which may introduce local steric strain within the binding pocket (Fig. 16).

## 4 Discussion

Olfaction is essential for the exploration and adaptation to the environment. It also serves as a valuable diagnostic marker for various neurological disorders and for monitoring disease progression (De Cleene et al., 2025, Naffaa, 2025). The absence of a well-established model of post-stroke Olfactory dysfunction has limited research in this condition. This investigation aimed to address this gap by characterizing the development of olfactory dysfunction in rats following 15 min of transient bilateral occlusion and reperfusion of the common and internal carotid arteries in rats. Furthermore, extracts of *Lantana camara* have been documented to exhibit antiepileptic, antihypertensive, and thrombolytic properties (Kandeda et al., 2022, Matta et al., 2015, Mujbaile et al., 2023). However, the effects of its aqueous extract on cerebral ischemia and associated olfactory dysfunctions have not been previously explored despite it ethnomedical usage in Cameroon. The study further sought to evaluate the efficacy of an aqueous *L. camara* leaf extract in ameliorating post-ischemic stroke sequelae and associated olfactory deficits.

Transient global cerebral ischemia-reperfusion in rats is an established experimental model, typically induced by methods such as two-vessel occlusion or bilateral common carotid artery occlusion combined with hypotension, which results in a temporary, severe reduction in cerebral blood flow (CBF) across the entire brain. This period of widespread hypoperfusion primarily causes selective neuronal vulnerability, leading to delayed neuronal death in highly susceptible regions, particularly the hippocampus, mimicking aspects of human cardiac arrest and subsequent brain injury (Oumer et al., 2021). The model employed in the current study represents a modification of the bilateral carotid occlusion approach. The CBF response in this model is reported as a triphasic pattern critical to post-ischemic injury. During the ischemic insult, CBF is severely reduced. Upon reperfusion, there is typically a brief phase of initial hyperemia where flow temporarily surges above baseline. This is quickly followed by the most damaging phase: delayed cerebral hypoperfusion, where CBF remains significantly for several hours to days, particularly in vulnerable regions like the hippocampus (Kumar et al., 2023, Liu et al., 2022, Melgar et al., 2002). This model is therefore reliable for inducing post-stroke neurological sequelae. In addition, by bypassing the compensatory retrograde perfusion provided by the external carotid branches, ICA occlusion ensures a consistent reduction in cerebral blood flow, leading to widespread tissue necrosis and reliable ischemic injury (Oumer et al., 2021).

Body temperature variation during an ischemic insult is one of the most powerful and well-documented determinants of neuropathological damage (Corbett and Thornhill, 2000, Dietrich et al., 1990). In the current study, analysis of body temperature dynamics during and immediately following the surgical procedure reveals a critical physiological difference between the sham and IR groups. The transient procedural hypothermia observed in both surgical groups, exacerbated in the IR group by the ischaemia-reperfusion event itself, is a known consequence of anaesthesia and surgical stress (Herrmann et al., 2003, Karaszewski et al., 2013). However, the subsequent trajectory observed in this study was highly significant: the IR group not only experienced a more severe initial hypothermia but also demonstrated a marked failure in homeostatic recovery, characterized by a transition to compensatory hyperthermia, a potentially detrimental outcome. This post-reperfusion hyperthermia represented a critical, self-reinforcing driver of secondary brain injury in stroke models, as even slight increases in core temperature can exacerbate excitotoxicity, oxidative stress, and neuroinflammation (Davis et al., 2023, Truettner et al., 2018). This contrasted sharply with the relatively stable, near-baseline thermal recovery observed in the sham group. This lack of homeostatic control was likely due to damage to thermosensitive nuclei (e.g., the preoptic area of the hypothalamus) (Kim et al., 2022), suggesting that the insult was widespread and severe enough to disrupt fundamental autonomic functions. Thus, the magnitude and duration of post-reperfusion hyperthermia were used as a prognostic biomarker for predicting the extent of eventual neurological and behavioral deficits, including the observed olfactory dysfunction.

This study establishes a clear pathophysiological link between behavioral deficits, biochemical disturbances, and histological damage following transient global cerebral ischemia-reperfusion. Behaviorally, IR rats exhibited profound neurological impairments, evidenced by elevated modified Neurological Severity Scores and significant locomotor deficits in open field tests (Langdon et al., 2008). Crucially, the buried food-seeking test revealed severe olfactory dysfunction, as indicated by increased latencies to locate food. The successful performance of all animals in the unburied trial served as a critical control. The ability of IR rats to retrieve visible food, despite a minor increase in latency, indicated that the primary deficit was olfactory in nature rather than sensorimotor or motivational. The persistence of these deficits over seven days suggested that endogenous regenerative capacities were insufficient to resolve the ischemic damage (Hwang et al., 2004, Nakashima et al., 1984). Biochemically, these behavioral failures correlated directly with a depletion of acetylcholine across the olfactory bulb, piriform cortex, prefrontal cortex, and hippocampus. ACh is vital for modulating neuronal excitability and synaptic plasticity; its reduction explained the inability of ischemic rats to identify or discriminate odors during memory tasks (Linster and Hasselmo, 2001, Tabassum and Haider, 2016). Furthermore, the biochemical profile revealed a state of intense oxidative stress (elevated MDA and nitrites; decreased GSH) and neuroinflammation (increased IL-1β and TNF-α). This oxidative-inflammatory surge represented a primary driver of cellular death during reperfusion (Espinós et al., 2020, Yao et al., 2019).

The histopathological profile observed in the IR group, encompassing neuronal loss, ghost cell and eosinophilic neuron formation, neuropil vacuolation, astrogliosis, and architectural disorganization, is consistent with the well-characterized cascade of excitotoxicity, oxidative stress, and neuroinflammation that underlies ischemia-reperfusion injury (Kuriakose and Xiao, 2020). Critically, the pattern of damage observed across the four examined regions reflects impairment of the olfacto-mnemonic axis, a functionally integrated circuit linking olfactory processing to memory consolidation and cognitive function. The olfactory bulb exhibited moderate neuronal loss, ghost cell formation, erythrocytic infiltration, and glomerular dystrophy; disruption at this level would be expected to compromise the fidelity of sensory input to downstream structures, including the piriform cortex and hippocampus, with which the olfactory bulb shares direct anatomical projections via the lateral olfactory tract (Leboucq et al., 2013, Silkis, 2023). The piriform cortex, which subserves both odor detection and olfactory memory, exhibited the most pronounced ghost cell burden alongside moderate vacuolation and tissue disruption, consistent with its high metabolic demand and particular vulnerability to ischemic insult (Sancandi et al., 2018, van Hartevelt and Kringelbach, 2015). The prefrontal cortex showed mild-to-moderate pathological changes, in keeping with its comparatively greater collateral vascular supply relative to limbic structures (Funahashi, 2017), while the hippocampus bore the greatest overall pathological burden, with the highest ghost cell score across all regions and severe neuronal loss in the CA1, CA3, and dentate gyrus subfields, a finding attributable to the high density of glutamate receptors, elevated metabolic activity, and limited antioxidant buffering capacity characteristic of this region (Nakashima et al., 1984, Silkis, 2023). Taken together, the concurrent damage across these four interconnected structures provides a compelling histopathological substrate for the observed learning, memory, and olfactory processing deficits.

Treatment with the aqueous extract of *Lantana camara* (AELC; 280 and 560 mg/kg) effectively addressed these pathological findings, significantly improving performance in novel odor recognition and spatial memory tests (Djeuzong et al., 2021, Djiogue et al., 2018), with effects that were dose-dependent and comparable in magnitude to those of minocycline (100 mg/kg) and piracetam (250 mg/kg), two pharmacological agents with well-established neuroprotective profiles in ischemia-reperfusion models. Minocycline exerts neuroprotection primarily through inhibition of microglial activation, suppression of matrix metalloproteinase activity, and attenuation of mitochondria-mediated apoptosis (Lu et al., 2025, Zhao et al., 2023), while piracetam enhances membrane fluidity, modulates AMPA receptor function, and improves cerebral microcirculation (Güngör et al., 2011, Kessler et al., 2000). The neuroprotective effects of AELC are mechanistically supported by concurrent biochemical observations demonstrating significant attenuation of oxidative stress, suppression of neuroinflammatory signaling, and restoration of cholinergic tone across the investigated brain regions. Ischemia-reperfusion injury triggers a rapid, self-amplifying burst of reactive oxygen species (ROS) upon reperfusion, driven by xanthine oxidase activation, mitochondrial electron transport chain dysfunction, and NADPH oxidase activity, promoting lipid peroxidation of neuronal membranes, protein carbonylation, and DNA strand breaks (Zhang et al., 2024). This collectively accelerating the progression from reversible ischemic injury to irreversible neuronal necrosis, the histological correlate of which is the ghost cell and eosinophilic neuron formation prominently observed in the IR group. The antioxidant activity of AELC would be expected to interrupt this cascade by scavenging ROS, chelating redox-active metal ions, and upregulating endogenous antioxidant enzyme expression, thereby reducing membrane damage and attenuating the ghost cell burden observed histologically (Dervash et al., 2025). Concurrently, microglial activation following ischemia-reperfusion promotes the release of pro-inflammatory cytokines including TNF-α and IL-1β, which amplify blood-brain barrier disruption, recruit peripheral immune cells, and directly induce neuronal apoptosis (Kuriakose and Xiao, 2020). The astrogliosis observed in the olfactory bulb and hippocampus of IR represents a histological hallmark of this neuroinflammatory response, reflecting both a reactive attempt at tissue repair and a contributor to the glial scar that impedes neuronal regeneration (Cerina et al., 2024, Langdon et al., 2008). The anti-inflammatory activity of AELC, consistent with the biochemically observed suppression of pro-inflammatory cytokine levels, likely underlies the marked reduction in astrogliosis and architectural disorganization evident in extract-treated groups.

Furthermore, the restoration of cholinergic tone by AELC in the investigated regions is particularly relevant to the functional integrity of the olfacto-mnemonic axis. Acetylcholine plays a pivotal role in hippocampal-dependent memory consolidation through modulation of long-term potentiation and gating of sensory inputs to cortical memory circuits (Ross et al., 2019, Schilit Nitenson et al., 2019). Ischemia-reperfusion injury is known to reduce cholinergic transmission through elevation of acetylcholinesterase activity, depletion of choline acetyltransferase, and loss of basal forebrain cholinergic neurons that project to the hippocampus and cortex (Alla et al., 2024, Decker and Duncan, 2020, Nikbakhtzadeh et al., 2025). The concurrent histological preservation of CA1, CA3, and dentate gyrus architecture in AELC-treated animals, together with restored acetylcholine levels, suggests that the extract may protect cholinergic afferents and their target neurons, thereby preserving the synaptic substrate required for olfactory memory processing. Collectively, the results demonstrate that *L. camara* confers neuroprotection by mitigating the biochemical cascades that lead to histological damage and subsequent behavioral impairment.

The phytochemical profiling of the AELC via LC-MS identified six bioactive compounds spanning diverse chemical classes. Among the identified constituents, alpha-pinene (a bicyclic monoterpenoid) exhibited the highest relative abundance, closely followed by the alkane tridecane and the steroid glycoside complex Charantin, which eluted at the highest retention time as a mixture of β-sitosteryl glucoside and 5,22-stigmasteryl glucoside. Moderate abundances were recorded for the flavonoid Salvigenin, the naphthoquinone Kigelinone, and the saturated fatty acid behenic acid. This phytochemical profile is broadly consistent with previously reported constituents of *L. camara* extracts(Chitapalli and Kemisetti, 2024; Dervash et al., 2025; Ezzat et al., 2020). The detection of inherently hydrophobic and volatile constituents such as alpha-pinene and tridecane in an aqueous matrix warrants methodological clarification. Their presence was attributable to the combined decoction-maceration extraction protocol employed. Indeed, boiling at 100°C thermally disrupts rigid plant cell walls, melts cuticular waxes housing tridecane, and ruptures glandular trichomes where volatile monoterpenoids such as alpha-pinene are stored, thereby releasing these compounds into the aqueous environment (Cao et al., 2025, Cetinkaya et al., 2025). During subsequent maceration, the abundant amphiphilic saponins and steroid glycosides intrinsic to *L. camara,* such as Charantin likely acted as endogenous emulsifiers, forming micelles that encapsulated these non-polar compounds and maintained them in stable micro-emulsion within the aqueous matrix(Rani and Tuteja, 2025). It should nonetheless be noted that the reported percentages reflect relative LC-MS chromatographic peak areas, which are influenced by compound-specific ionization efficiencies under the analytical conditions applied, rather than absolute mass concentrations in the extract.

The *in silico* profiling of the compounds identified in the AELC provides a clear roadmap for their pharmacological development. Based on the integration of Lipinski’s Rule of Five and ProTox−3.0 toxicity predictions, Salvigenin, Kigelinone, and alpha-pinene emerge as the most promising scaffolds. Their lack of predicted organ toxicity combined with high membrane permeance makes them ideal lead for systemic anti-inflammatory therapies. While Kigelinone demonstrates excellent drug-likeness, the predicted potential for hepatotoxicity suggests that future *in vivo* studies should prioritize liver function monitoring and dosage optimization to mitigate oxidative stress. Based on these predictions, the observed neuroprotective effects are most likely attributed to Salvigenin, alpha-pinene, and Kigelinone. These compounds satisfied Lipinski's Rule of Five and exhibited high predicted membrane permeance, suggesting they can efficiently cross biological barriers to reach neural targets. Alpha-pinene confers neuroprotection by deploying anti-inflammatory and anti-apoptotic mechanisms in an animal model of focal cerebral ischemia-reperfusion (Khoshnazar et al., 2020). Salvigenin acts as a neuroprotective agent by effectively inhibiting both apoptosis and oxidative stress within the hippocampus and cortex of rats subjected to amyloid-beta-induced toxicity (Esfandabadi et al., 2013). Kigelinone further contributes to the extract's profile by exhibiting documented antioxidant properties, anti-inflammatory effects, and acetylcholinesterase inhibitory activity (Nabatanzi et al., 2020). Although charantin is predicted to exhibit low blood-brain barrier (BBB) permeability, it may nonetheless contribute to neuroprotection through indirect pathways or metabolic biotransformation. The literature reveals that the phytosterol Stigmasterol exerts its neuroprotective effects by upregulating the endogenous antioxidant defense system and modulating cellular signaling cascades, notably through the inhibition of autophagy activation via the AMPK-mTOR and JNK pathways (Sun et al., 2019). Similarly, the related phytosterol β-sitosterol was shown to mitigate cognitive impairment induced by AlCl₃ by reducing cerebral oxidative stress, restoring cholinergic function, and suppressing the expression and enzymatic activity of key inflammatory and regulatory targets, including glycogen synthase kinase−3β, Rho kinase, lipoxygenase−5, TNF-α, cyclooxygenase−2, and the Na⁺/K⁺-ATPase pump (Sajad et al., 2025). Further chemical analyses should prioritize the quantification of these identified active components.

The docking procedure was driven by significant in *vivo* behavioral findings. In deed treatment with *Lantana camara* extracts resulted in a marked enhancement of olfacto-cognitive performance in the current study. Given that both olfactory processing and cognitive functions are heavily modulated by the cholinergic system, specifically through the high-affinity nicotinic acetylcholine receptors (nAChRs) expressed in the hippocampus and olfactory bulb *(*Dineley et al., 2015), we hypothesized that the extract contained compounds that can directly interact with this receptor. The α7 nAChR is a primary candidate for this effect due to its role in synaptic plasticity and sensory gating (Uwada et al., 2020). However, because orthosteric activation often leads to rapid desensitization, we also hypothesized that the observed cognitive improvement might stem from allosteric modulation. To investigate this, we utilized the cryo-EM structure of the human α7 nAChR (PDB ID: 7KOX) to perform a curvature-based blind docking analysis. The identification of the transmembrane domain vestibule as the primary binding site for Kigelinone and Salviginin, consistent with the established binding profile of the reference modulator PNU−120596 (Uwada et al., 2020). This molecular docking analysis suggests that Kigelinone and Salviginin may interact with a putative allosteric site of the receptor in a pose consistent with positive modulation; however, this remains a computational prediction and requires experimental validation through electrophysiology, binding assays, and functional studies before any mechanistic classification can be established. Structural analysis reveals that Kigelinone’s superior affinity is primarily facilitated by a high-strength hydrogen bond with Ala262 in the M2 helix of Chain A, a residue that is crucial for lining the ion conduction pore and regulating channel gating (Burke et al., 2024). This robust interaction, alongside a stabilizing network of π-anion and π-π T-shaped stacking with residues such as Glu172 and Tyr210, suggests a molecular glue mechanism that stabilizes the receptor’s active conformation and potentially attenuates the rapid desensitization inherent to α7 nAChRs (Noviello et al., 2021). Conversely, while Salviginin maintains a stable interaction profile, its slightly lower affinity is likely influenced by an unfavorable contact with Lys45, a key residue in the β1-β2 loop involved in signal transduction coupling(Amiri et al., 2005; Wang et al., 2025). While PNU-120596 relies on this singular polar anchor and extensive distal hydrophobic interactions, Kigelinone demonstrated a superior binding profile by establishing a multi-point hydrogen-bonding network (4 bonds). These molecular insights highlight the promise of *L. camara* phytochemicals as neuroprotective scaffolds for the treatment of other neurodegenerative disorders where α7 nAChR hypofunction is a hallmark pathology (Decker and Duncan, 2020). While PNU−120596 served as a computational α7 nAChR-specific reference, future studies should validate these findings with *in vitro* functional assays using selective α7 nAChR agonists and antagonists to confirm receptor engagement.

Several limitations of the present study warrant acknowledgement. First, the histological analysis was conducted on four animals per group (n = 4), a sample size appropriate for a preliminary, descriptive investigation but insufficient to support definitive quantitative conclusions or robust statistical inference. Future studies should employ design-based stereology with adequately powered cohorts (n ≥ 5 per group) to provide unbiased quantitative estimates of neuronal density, olfactory bulb volume, and glial cell populations using established methods such as the optical fractionator or Cavalieri estimator in place of the semi-quantitative ordinal scoring system employed here. Second, the absence of immunohistochemical characterization represents a further methodological constraint. The pathological processes identified on haematoxylin and eosin staining would benefit from confirmation using established cellular markers such as NeuN (neuronal nuclei), Iba−1 (microglial activation), and GFAP (reactive astrogliosis). Incorporation of markers for neuronal apoptosis (e.g. cleaved caspase−3, TUNEL), neuronal survival, and neurotrophic factor signaling (e.g. BDNF, TrkB) in future work would allow more precise mechanistic characterization of both the ischemic injury and the neuroprotective response to AELC. Third, the scope of behavioral assessment should be expanded in future investigations to comprehensively map post-ischemic deficits along the olfacto-mnemonic axis. In addition to the standard memory and locomotor paradigms employed in the present study, a broader battery including odor detection threshold testing, olfactory discrimination tasks, and olfactory habituation-dishabituation protocols would enable more granular characterization of olfactory dysfunction following stroke and a fuller evaluation of the therapeutic reach of *L. camara* extract. Fourth, the cholinergic mechanism underlying the observed increase in acetylcholine levels in the examined brain regions requires further elucidation. Future studies should determine whether this effect reflects enhanced choline acetyltransferase activity, protection of basal forebrain cholinergic neurons, or a combination of these mechanisms, using targeted enzymatic assays, Western blotting, and receptor binding studies. Fifth, cerebral blood flow was not quantified within the affected brain regions in the present study. Incorporating perfusion imaging or laser Doppler flowmetry in future experiments would provide objective validation of the ischemia-reperfusion surgical model and would allow the vascular contributions to AELC-mediated neuroprotection to be distinguished from direct neuronal or glial effects. Sixth, the phytochemical characterization of the aqueous extract, while identifying the major constituent compound classes by LC-MS, was limited to qualitative detection without absolute quantification of individual marker compounds. In the absence of authenticated external reference standards run alongside the extract, only semi-quantitative relative abundance estimates could be obtained. This precludes direct pharmacokinetic interpretation of the dose-response relationships observed and limits comparability with studies reporting standardized, quantified extracts. Furthermore, as the extract was prepared and administered as a single batch, inter-batch consistency was not assessed, an evaluation that would be a prerequisite for any translational application. Future studies should address these limitations through bioactivity-guided fractionation coupled with quantitative LC-MS/MS analysis using authenticated reference standards, enabling both absolute quantification of marker compounds and establishment of batch-to-batch reproducibility as a prerequisite for pharmaceutical development.

## 5 Conclusion

Stroke remains a leading cause of mortality and long-term disability globally, yet post-stroke olfactory dysfunction remains without an approved pharmacological intervention, representing a critical unmet clinical need particularly acute in developing nations where specialized stroke care is severely limited. The present study addresses this gap through a multidisciplinary investigation that achieved two primary objectives: the establishment and validation of a reproducible rat model of post-stroke olfactory dysfunction using a 15-minute global cerebral ischemia-reperfusion protocol, and the preclinical evaluation of the aqueous extract of *Lantana camara* (AELC) as a candidate therapeutic intervention. The post-ischemic olfacto-mnemonic deficit was underpinned by cholinergic depletion, compounded by a self-amplifying cascade of oxidative stress and neuroinflammation across the olfactory bulb, piriform cortex, prefrontal cortex, and hippocampus, four anatomically interconnected structures whose concurrent pathological involvement provided a compelling histopathological substrate for the behavioral and biochemical deficits observed. AELC at 280 and 560 mg/kg effectively attenuated these alterations, restoring cholinergic tone, suppressing neuroinflammatory signaling, reducing oxidative damage, and preserving neuronal microarchitecture across all examined regions, with effects comparable to those of the reference compounds minocycline and piracetam. These neuroprotective effects are consistent with the previously documented antioxidant, anti-inflammatory, antihypertensive, and thrombolytic properties of *L. camara*, and extend them to the specific domain of post-stroke olfactory and cognitive recovery. Phytochemical profiling identified six bioactive constituents spanning diverse chemical classes, among which alpha-pinene, Salvigenin, and Kigelinone emerged as the most pharmacologically promising leads based on *in silico* drug-likeness screening, toxicity prediction, and molecular docking analyses. Both compounds were identified as potential type II positive allosteric modulators of the α7 nicotinic acetylcholine receptor, with Kigelinone exhibiting predicted binding interactions at a site topographically consistent with known allosteric modulators of the α7 nAChR, a mechanistic hypothesis that warrants validation through functional electrophysiological and binding studies, providing a plausible molecular basis for the observed cholinergic restoration. Collectively, these findings validate the ethnomedicinal use of *L. camara* aqueous leaf extract documented in southern Cameroon and related African and Asian traditions, and position AELC as a pharmacologically rational candidate for the development of plant-derived allosteric modulators targeting post-stroke olfactory and cognitive dysfunction. Future research should prioritize bioactivity-guided isolation and absolute quantification of theses compound, together with their systematic preclinical and clinical validation, to address the urgent need for accessible, biofriendly neuroprotective strategies in stroke recovery and cholinergic-linked neurodegenerative disorders, particularly in under-resourced healthcare settings where conventional therapeutic options remain critically constrained.

## CRediT authorship contribution statement

**Symphorien Talom Mabou:** Writing – review & editing, Writing – original draft, Visualization, Validation, Methodology, Investigation, Conceptualization. **Antoine Kavaye Kandeda:** Writing – review & editing, Writing – original draft, Visualization, Validation, Supervision, Conceptualization. **Claude Danielle Bilanda:** Writing – review & editing, Writing – original draft, Visualization, Validation, Supervision, Methodology, Conceptualization. **Ferdinand Tameu Meuladje:** Writing – original draft, Software, Resources, Investigation. **Bernes Rivaldo Tadah Kahou:** Writing – original draft, Software, Resources, Methodology, Investigation, Conceptualization.

## Ethical statement

All animal experiments were conducted in accordance with the National Institutes of Health Guide for the Care and Use of Laboratory Animals / European Union Directive 2010/63/EU. The experimental protocol was reviewed and approved by the Institutional Animal Care and Use Committee of University of Yaoundé 1. All procedures involving the animals adhered to the ethical guidelines established by the Institutional Ethics Committee of the Cameroon Ministry of Scientific Research and Technological Innovation (Reg. no. FWA-IRD 0001954, 04/09/2006). All efforts were made to minimize animal suffering and to reduce the number of animals used. Furthermore, the study design and manuscript preparation comply with the ARRIVE guidelines for reporting in vivo experiments.

## Funding sources

This research did not receive any specific grant from funding agencies in the public, commercial, or not-for-profit sectors.

## Declaration of Competing Interest

The authors declare that they have no known competing financial interests or personal relationships that could have appeared to influence the work reported in this paper.
